# Localization of Candidate Regions Maintaining a Common Polymorphic Inversion (2La) in Anopheles gambiae

**DOI:** 10.1371/journal.pgen.0030217

**Published:** 2007-12-07

**Authors:** Bradley J White, Matthew W Hahn, Marco Pombi, Bryan J Cassone, Neil F Lobo, Frederic Simard, Nora J Besansky

**Affiliations:** 1 Center for Global Health and Infectious Diseases, Department of Biological Sciences, University of Notre Dame, Notre Dame, Indiana, United States of America; 2 Department of Biology and School of Informatics, Indiana University, Bloomington, Indiana, United States of America; 3 Istituto Pasteur-Fondazione Cenci Bolognetti and Dipartimento di Scienze di Sanità Pubblica, Università di Roma “La Sapienza,” Rome, Italy; 4 Institut de Recherche pour le Développement, Unité de Recherche R016, Montpellier, France; 5 Organisation de Coordination pour la Lutte contre les Endémies en Afrique Centrale, Yaounde, Cameroon; Fred Hutchinson Cancer Research Center, United States of America

## Abstract

Chromosomal inversion polymorphisms are thought to play a role in adaptive divergence, but the genes conferring adaptive benefits remain elusive. Here we study 2La, a common polymorphic inversion in the African malaria vector Anopheles gambiae. The frequency of 2La varies clinally and seasonally in a pattern suggesting response to selection for aridity tolerance. By hybridizing genomic DNA from individual mosquitoes to oligonucleotide microarrays, we obtained a complete map of differentiation across the A. gambiae genome. Comparing mosquitoes homozygous for the 2La gene arrangement or its alternative (2L+^a^), divergence was highest at loci within the rearranged region. In the 22 Mb included within alternative arrangements, two ∼1.5 Mb regions near but not adjacent to the breakpoints were identified as being significantly diverged, a conclusion validated by targeted sequencing. The persistent association of both regions with the 2La arrangement is highly unlikely given known recombination rates across the inversion in 2La heterozygotes, thus implicating selection on genes underlying these regions as factors responsible for the maintenance of 2La. Polymorphism and divergence data are consistent with a model in which the inversion is maintained by migration-selection balance between multiple alleles inside these regions, but further experiments will be needed to fully distinguish between the epistasis (coadaptation) and local adaptation models for the maintenance of 2La.

## Author Summary

A chromosomal inversion occurs when part of the chromosome breaks, rotates 180 degrees, and rejoins the broken chromosome. The result is a chromosome carrying a segment whose gene order is reversed. Whereas the physical rearrangement itself may have no direct consequences on gene function, recombination between alleles in the rearranged and wild type segments is suppressed. If multiple alleles inside the inverted or original orientations are well adapted to contrasting environmental conditions, suppressed recombination provides a mechanism to keep beneficial allelic combinations from being shuffled between different genetic backgrounds. Working with wild populations of flies, Dobzhansky provided the first evidence that selection was key to maintaining inversion polymorphism. Subsequently, examples of inversion polymorphisms under selection in other organisms have been found, notably in the mosquito that transmits most cases of human malaria, Anopheles gambiae. However, the genes or gene regions conferring fitness advantages have yet to be discovered. In this study, the authors used modern genomics tools to map such regions in an inversion at an unprecedented level of detail, and show that these regions are likely to be responsible for the maintenance of the inversion polymorphism in natural populations. This study lays the groundwork for future efforts to identify the genes themselves and their role in adaptation.

## Introduction

Dobzhansky's studies of chromosomal inversion polymorphisms in natural populations of *Drosophila* provided the first evidence that selection played an indispensable role in their maintenance, helping to spark the neo-Darwinian synthesis [1,2]. More recent studies implicate selection in maintaining inversion polymorphisms in a diversity of eukaryotes, including humans [3–6]. A mechanism thought to facilitate their maintenance is reduced recombination. In inversion heterozygotes (heterokaryotypes), recombination between alternate arrangements may be inhibited both by asynapsis and because single crossovers within an inversion loop result in aneuploid meiotic products [7]. Such reduced recombination binds together favorably interacting genes (coadapted gene complexes) and/or multiple genes that are individually adapted to local conditions, and stabilizes them against gene exchange with migrants from other genetic backgrounds [1,8]. Stabilization of these allelic combinations allows the inversion to establish and spread, and consequently organisms can become adapted to highly divergent environmental conditions. Although selection has been invoked repeatedly to explain the maintenance of chromosomal inversions—and in some cases associated phenotypic traits have been identified [4]—the genes or regions involved remain elusive.

If a small subset of genes within an inversion were under selection and there was no gene flux at all between arrangements (sensu [9]), it would be impossible to identify specific genes or even regions affected by selection. Fortunately, inhibition of gene exchange between alternative gene arrangements is not absolute. Except near inversion breakpoints, gene conversion is unaffected and double crossovers can result in balanced recombinant gametes [10,11]. Working together, both recombinational processes (gene flux, [10]) gradually break up linkage disequilibrium within arrangements and homogenize sequence variation between them, unless countered by selection. The interaction of gene flux and selection is expected to produce a mosaic of more- and less-differentiated regions inside the inversion and away from breakpoints, exactly the pattern observed from some molecular studies of inversion polymorphisms in natural populations (e.g.*,* [12,13]). These observations suggest that regions affected by selection can be identified, if not the precise genes and mutations involved. It is expected that such regions will be in significant linkage disequilibrium with the inversion and with each other even when they are not adjacent. However, a neutral explanation for patterns of linkage disequilibrium also exists: regions significantly associated with the inversion polymorphism may simply represent historical remnants of the genetic background upon which mutations arose. These alternative hypotheses can be tested using estimates of the rate of genetic exchange in heterokaryotypes and the age of inversion polymorphism.

Mapping of divergent regions between chromosomal arrangements is a prerequisite to identifying candidate genes under selection and ultimately elucidating the molecular basis of adaptations conferred by inversions. The reduced level of recombination in heterokaryotypes renders a traditional QTL (quantitative trait locus) mapping approach impractical. Instead, genomic scans of nucleotide divergence in natural populations take advantage of recombination over many generations. Previous scans for divergence and linkage disequilibrium in inversions have been hindered by the low resolution afforded by limited numbers of genetic markers. Application of modern genomics tools to the classical study of inversions has the potential to both accelerate and refine the mapping of diverged regions. Recent studies have demonstrated the utility of Affymetrix GeneChip arrays as high density genetic markers [14–17]. Emitted fluorescence from individual probes on the array directly correlates with the sequence similarity between hybridized DNA and the probe. In this manner, divergence between two genetic classes (e.g., alternative chromosomal orientations) can be examined at high resolution. Using this technique, we examined genic differentiation between individual Anopheles gambiae mosquitoes bearing alternate chromosomal arrangements—2La and 2L+^a^—on the left arm of the second chromosome, as a first step in identifying candidate regions maintaining inversion 2La in natural populations.

The 2La inversion system in A. gambiae is of interest not only as a model for understanding the adaptive role of inversions, but also for its epidemiological importance. A. gambiae is the most proficient vector of human malaria in the world, causing more than one million malaria-related deaths in sub-Saharan Africa each year [18]. Abundant inversion polymorphisms on chromosome 2 appear to play a key role in the ecological success of this species, as different inversion combinations are nonrandomly associated with both natural and anthropogenic environmental heterogeneities [3,19,20]. Inversion 2La is of particular interest and significance. First, it is the only inversion polymorphic on 2L, simplifying its analysis. Second, this inversion was acquired from an arid-adapted sibling species, A. arabiensis, by introgressive hybridization [3,21,22]. The nonrandom association of 2La with degree of aridity points to the adaptive value of this polymorphism in A. gambiae. In many different locations across Africa, 2La frequency exhibits strong and stable geographic clines from near fixation in arid zones to complete absence in humid rainforests [23–26]. Similarly, its frequency varies seasonally and microspatially according to patterns of rainfall and microclimate. Thus mosquito carriers of 2La are more likely than carriers of 2L+^a^ to rest inside houses at night where a saturation deficit exists, affecting the probability of vector–human contact at peak blood feeding times (reviewed in [27]). From the standpoint of epidemiology and human health, the 2La polymorphism has increased malaria transmission by A. gambiae across diverse ecoclimatic zones and it could mitigate the efficacy of control measures that assume uniform indoor resting and biting behavior, such as bednets and indoor insecticide (or fungicide) application. Its study is facilitated by several recent developments, including a completely sequenced reference genome [28] and a resulting Affymetrix GeneChip array already proven to be an effective population genomics tool [16]. Furthermore, the breakpoints of the 2La inversion have been characterized molecularly [29] and the rate of genetic exchange on 2L between 2La/+^a^ heterozygotes has been estimated in laboratory crosses [30].

In the following we address the selective maintenance of the 2La inversion polymorphism by multiple experiments. (1) We applied the Affymetrix GeneChip to map, at unprecedented resolution, highly diverged regions between alternate arrangements of a chromosomal inversion (2La). (2) We validated the principal microarray findings by targeted DNA sequencing, and used the resulting nucleotide polymorphism data to ask whether the introgressed chromosomal arrangement rose to its current frequency via adaptive natural selection. (3) We used known recombination rates in inversion heterokaryotypes to assess the likelihood that linkage disequilibrium between diverged regions and the inversion is maintained by selection.

## Results

### Comparative Genome Hybridization

The *Anopheles* probes on the Affymetrix GeneChip Anopheles/Plasmodium Array were designed mainly from the reference A. gambiae genome assembled from the chromosomally standard (uninverted) PEST strain [28]. The 25 bp probes (11 per transcript) interrogate ∼14,900 putative transcripts predicted from an early gene build (GeneBuild 2, 2003). From this core set of probes, those with more than one perfect match or with single nucleotide mismatches elsewhere in the genome were excluded from the analysis. The remaining 151,213 unique probes were distributed across the genome roughly in proportion to chromosome arm length. The 49 Mb chromosome 2L was represented by 33,892 probes, of which 13,984 mapped within the 22 Mb 2La inversion.

To minimize the contribution of ecological or geographic diversity to genetic variation, samples of A. gambiae homozygous for alternative 2L arrangements (2La/a and 2L+^a^/+^a^) were collected simultaneously from one village in central Cameroon where 2La is highly polymorphic and inversion heterozygotes are common (the 2La frequency was 46% in our 2005 sample of 70 mosquitoes, and in samples collected from the same village in 2002–2003 its frequency was 39%; F. Simard, unpublished data). The specimens used in this study were all identified as the S molecular form, one of two assortatively mating incipient species of A. gambiae [31]. Three of five 2La/a specimens were polymorphic for inversions on 2R; all other specimens carried the 2R standard arrangements. Labeled genomic DNA from each of five 2La/a and 2L+^a^/+^a^ mosquitoes was hybridized to individual arrays (ten in total) to map nucleotide divergence, measured in terms of single feature polymorphisms (SFPs). SFPs were defined as probes whose hybridization intensities were significantly different between the five carriers of each 2L arrangement, as determined by two-tailed t-tests with a threshold of *p* < 0.01 [16]. A significant difference in hybridization intensity between samples reflects underlying differences in the target nucleotide sequences interrogated by the probes on the array.

**Figure 1:**
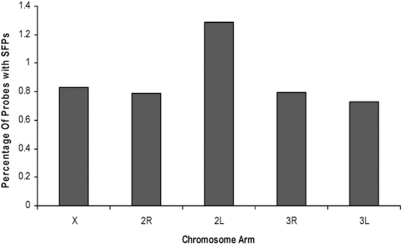
Divergence by Chromosome Arm between Homozygous Carriers of 2La and 2L+^a^ Arrangements

**Figure 2:**
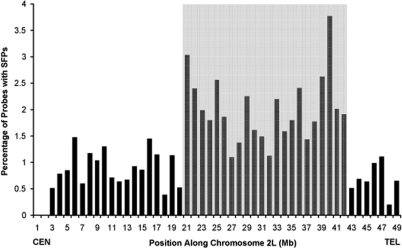
Divergence Across Chromosome 2L between Homozygous Carriers of 2La and 2L+^a^ Arrangements Shaded area represents the rearranged region. CEN, centromere; TEL, telomere.

Genome-wide, 1,352 probes (0.89%) were SFPs between 2La- and 2L+^a^-carriers. Of these, 444 were found on 2L, 283 of which were found in the rearranged region. The proportion of SFPs was notably higher on 2L (1.29%) than across the other four chromosome arms (0.79%; *p* < 1×10^−21^), which show consistent levels of differentiation (Figure 1). Along 2L, SFPs were distributed disproportionately within as compared to outside the rearranged region (1.98% versus 0.80%; *p* < 1×10^−20^; Figure 2). Indeed, the level of differentiation on 2L outside the rearranged region was indistinguishable from that of the other four chromosome arms (*p* = 0.75).

The distribution of putative SFPs on 2L was also explored by implementing a hidden Markov model (HMM) to identify differentiated and homogenized regions independent of a priori information about 2La breakpoint locations (cf. [16]). The HMM identified a 22 Mb diverged region corresponding almost precisely to the rearranged region, beginning at the first probe inside the proximal breakpoint and ending only ∼386 kb (243 probes) beyond the distal breakpoint.

**Figure 3:**
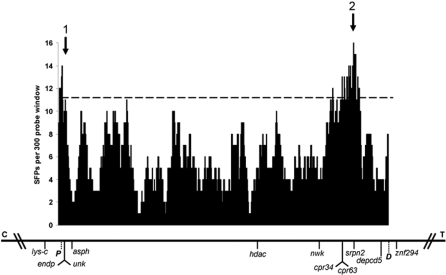
Sliding Window Analysis of Divergence between the Breakpoints of the 2La and 2L+^a^ Arrangements Horizontal dashed line is the significance threshold, at 11 SFPs per window. Arrows labeled “1” and “2” point to significant clusters of SFPs. Shown below the plot is a schematic representation of chromosome 2L (horizontal line) indicating the relative positions of 11 loci that were sequenced. P and D represent the proximal and distal breakpoints and are indicated by dashed vertical lines; C and T represent the centromeric and telomeric ends of 2L.

To test for regional clustering of SFPs between the breakpoints of the rearrangement, a sliding window analysis was performed that revealed two regions in which the observed number of SFPs was greater than expected by chance (Figure 3). The first (proximal) cluster (*p* = 0.001) extends ∼1.0 Mb, approximately from 2L coordinates 21.1–22.1 Mb and as measured from its midpoint, ∼1.1 Mb from the proximal breakpoint (labeled “1” in Figure 3). Though the boundaries of the clusters are necessarily imprecise, the first cluster spans roughly 32 genes and contains 17 SFPs. The second (distal) cluster (*p* < 1×10^−5^) extends between coordinates ∼38.8–40.5 Mb, ∼2.5 Mb from the distal breakpoint (labeled “2” in Figure 3). The 63 SFPs in this ∼1.7 Mb cluster span approximately 178 genes. Both clusters remained significant (*p* < 0.05) after correction for the number of windows tested. The genes predicted in each cluster according to Genebuild AgamP3.4 (http://agambiae.vectorbase.org/index.php) are listed in Supplementary Tables S2 and S3.

### DNA Sequence Divergence and Gene Flux

The microarray analysis suggested two patterns. Most striking was heightened divergence between the rearranged region and little elsewhere on 2L. In addition, two significant clusters of SFPs inside the rearrangement were found near, but not directly adjacent to, its proximal and distal ends. Because of the limited sample sizes in the microarray analysis, we sought to confirm and extend these results by targeted sequencing of an additional 24–34 chromosomes carrying each gene arrangement, sampled from the same Cameroon population of A. gambiae. Eleven genes were chosen based on their location within or outside of the rearranged region (Figure 3; Table 1). Two were located ∼1 Mb outside of the proximal and distal breakpoints; inside, one was located centrally, one within the proximal cluster, three within the distal cluster, and two just outside of and flanking each cluster. Wherever possible, the corresponding genes were also sequenced from 2–6 chromosomes of two sibling species: a sympatric population of A. arabiensis (fixed for 2La) and an allopatric population of A. quadriannulatus (fixed for 2L+^a^). We used three approaches to explore whether DNA sequences supported the microarray results: comparing numbers of shared versus fixed differences between chromosomal arrangements, summary statistics of nucleotide differentiation, and gene tree reconstruction.

**Table 1:**
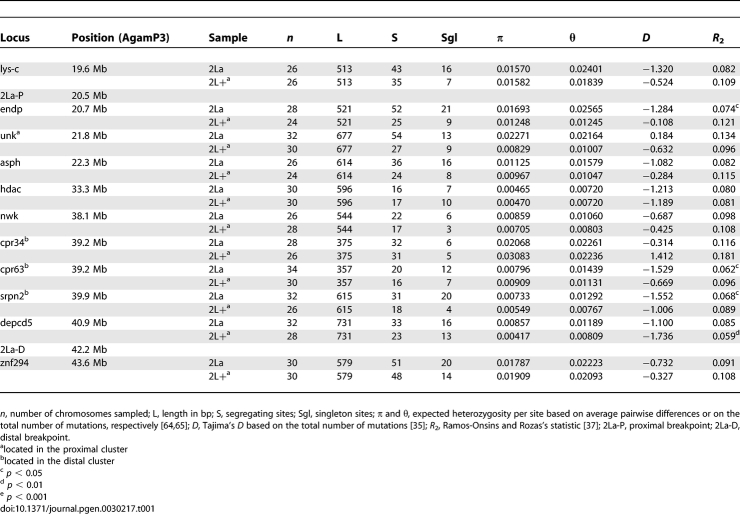
Polymorphism Statistics for 11 Genes Located on Chromosome 2L in A. gambiae

The numbers of polymorphisms shared between alternative arrangements in A. gambiae was high at the two genes outside the rearranged region (24 and 31), while the corresponding values for genes inside were much lower (ranging from 1–9) (Table 2). A small proportion of all shared polymorphisms may be due to recurrent mutation (Table 2; [32]), but most are shared because of gene flux (see below). As expected, the number of fixed differences followed a trend opposite to that of shared polymorphisms. No fixed differences occurred outside the rearranged region; inside, five of nine genes had fixed differences. The three genes nearest the proximal breakpoint all show relatively high numbers of fixed differences, even a gene distal to the proximal cluster (*asph*). These data therefore indicate that the boundaries of the proximal cluster were likely underestimated, though sequences at the breakpoint are expected to show fixed differences because of low levels of gene flux (see below). At the other end of the rearranged region, two of the three genes within the distal cluster show fixed differences, while no fixed differences were observed in either of the flanking genes, not even the one nearest the distal breakpoint. The ratio of fixed differences to shared polymorphisms for three genes within the distal cluster (*cpr34*, *cpr63*, *srpn2*; 9:14) was significantly higher than that for three genes outside and flanking the region (*hdac*, *nwk*, *depcd5*; 0:21; Fisher's exact test, *p* = 0.001). Because this test was conducted post hoc the results should be interpreted with some caution.

**Table 2:**
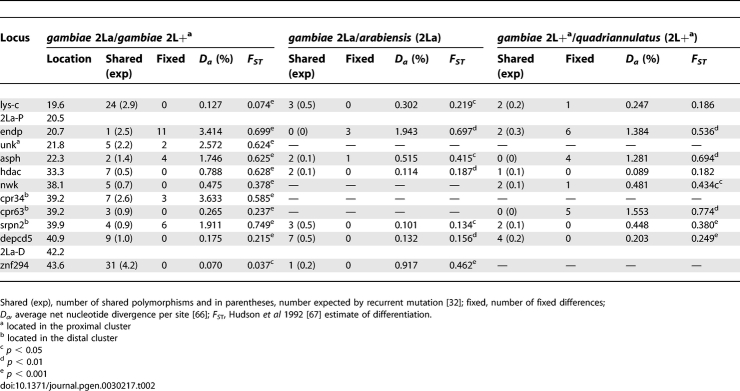
DNA Divergence and Gene Flow at 11 Genes on Chromosome 2L in *A. gambiae sl*

Differentiation between arrangements was also gauged by estimating *F_ST_* values and net divergence along 2L. Pairwise *F_ST_* values for all loci on 2L were significantly different from zero, but values within the rearranged region were roughly an order of magnitude larger than those in collinear regions (Table 2). Net divergence (*D*_a_) followed the same general pattern, with far greater levels of divergence observed inside the rearranged region. The average number of net nucleotide substitutions per site was 1.7% between arrangements and only 0.1% outside them. Moreover, those genes showing the highest levels of divergence were located in and around the proximal cluster and—with the exception of *cpr63*—inside the distal cluster. The lowest level of divergence was measured at *depcd5* nearest the distal breakpoint.

**Figure 4:**
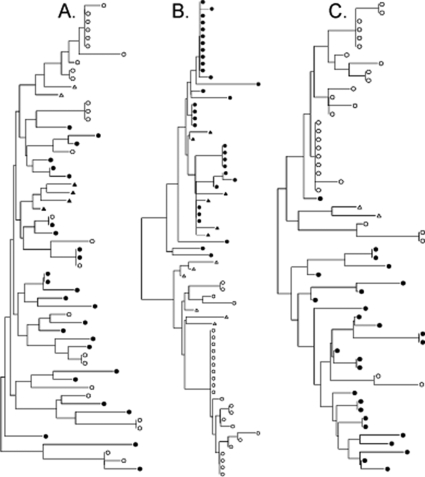
Representative Neighbor-Joining Gene Trees Reconstructed Using Sequences from Collinear or Rearranged Regions of Chromosome 2L Trees are drawn to scale, with branch lengths in units of substitutions per site. Taxon markers: black circle, A. gambiae 2La arrangement; black triangle, A. arabiensis (2La); white circle, A. gambiae 2L+^a^ arrangement; white triangle, A. quadriannulatus (2L+^a^). (A) *lys-c*; (B) *srpn2*; (C) *nwk*.

Gene trees reconstructed from sequences at each locus yielded three basic patterns that were consistent with those that emerged from measures of divergence and fixed/shared variation (Figure 4). Owing to recombination, these branching patterns do not represent the exact evolutionary history of the genes sampled, but they do portray contrasting pictures of the extent of genetic exchange between arrangement classes. The first pattern, exemplified by *lys-c* in Figure 4A, shows the complete intertwining of sequences from inverted and standard arrangements, as expected if gene exchange has been frequent. This pattern was shared by both genes located outside of the inversion, as well as two inside at the distal end: *cpr63* (in the distal cluster) and *depcd5*. These latter two genes showed the lowest level of net nucleotide divergence of any genes inside the inversion and correspondingly reduced *F_ST_* values. The second pattern, common to five genes within the inversion (*asph*, *endp*, *srpn2*, *unk* and *cpr34*), shows reciprocally monophyletic 2La and 2L+^a^ sequences, as expected if they are largely isolated. Three of four genes sampled from both clusters showed this pattern, including the *srpn2* gene illustrated in Figure 4B. Two remaining genes inside the inversion (*hdac* and *nwk*) gave a third pattern indicative of limited gene exchange, such that a single sequence of one arrangement clustered together with sequences of the opposite arrangement as illustrated for *nwk* in Figure 4C).

A notable feature of all gene trees where outgroup sequences were available was the embedding of A. arabiensis (2La) and A. quadriannulatus (2L+^a^) sequences inside of A. gambiae 2La and 2L+^a^ clades, respectively. A. gambiae is considered to have evolved from an A. quadriannulatus-like ancestor in a recent human-influenced speciation event in the central African rain forest [33]. If so, it would have carried only 2L+^a^, as A. quadriannulatus does. Although the 2La arrangement is ancestral in the A. gambiae sibling species complex [29,33], 2La likely passed into A. gambiae subsequent to the emergence of this species, following contact and genetic introgression with A. arabiensis [3,21,22]. Further evidence of the close genetic relationship between the same arrangement of 2L in different species can be seen from the contrasts presented in Table 2. In the rearranged region, greater differentiation exists between alternative arrangements within A. gambiae than between the same arrangement from different species. The opposite is true for collinear regions: differentiation is greater between species than within A. gambiae, due to free recombination in the latter.

### DNA Sequence Polymorphism

Given that deeper sequencing of both chromosomal arrangements confirmed the existence of two highly differentiated clusters of genes within the rearranged part of 2L, we next asked whether there was any signature of selection in these clusters or on the 2La arrangement. The presumed recent introgression of 2La is inconsistent with a long-term balanced polymorphism. If this newly invading inversion was subject to strong directional selection in its rise to its current frequency (∼46%)—and this selection occurred in the recent enough past—a signature of selection on the level and frequency of nucleotide polymorphism should be evident. We used the sequence data collected from the 11 loci in and around the inversion to detect such a pattern.

Levels of nucleotide diversity in the rearranged region (calculated from the two separate samples of 2La and 2L+^a^ chromosomes) were lower than in collinear regions (π: 1.11% versus 1.71%, Wilcoxon Rank-Sum test with 1-tail, *p* = 0.03; θ: 1.34% versus 2.14%, *p* < 0.02), as expected if there has been recent directional selection in the inversion. However, contrary to the expectation of the selective sweep hypothesis, levels of diversity within the inversion are highest within ∼1 Mb of the proximal breakpoint and generally decline moving distally (Table 1). The one exception to this pattern of declining heterozygosity is high levels of polymorphism in the *cpr34* gene in the distal cluster.

A second prediction of the selective sweep hypothesis is that 2La chromosomes should contain less polymorphism than 2L+^a^ chromosomes because of their more recent common ancestry. This pattern was not found: in fact, average levels of nucleotide diversity were slightly higher in 2La than in 2L+^a^ arrangements, though the difference was only significant when diversity was estimated from the number of segregating sites (π: 1.21% and 1.02%, Wilcoxon Signed-Rank Test, *p* < 0.20; θ: 1.59% and 1.09%, *p* < 0.01). In addition, HKA tests [34] comparing A. gambiae 2La with A. quadriannulatus (2L+^a^), and A. gambiae 2L+^a^ with A. arabiensis (2La) across loci from rearranged and collinear regions were not significant for either comparison (χ^2^ = 4.54, *p* < 0.96 and χ^2^ = 9.82, *p* < 0.40, respectively). These test results indicate that there is not significant heterogeneity in levels of diversity relative to divergence between rearranged and collinear regions, consistent with the absence of recent hitchhiking on 2L and the lack of major differences in mutation rate between lineages.

Two within-locus tests of deviation from the neutral-equilibrium model were conducted separately for 2La and 2L+^a^ arrangements. Similar to previous sequence surveys of A. gambiae (e.g., [30]), Tajima's *D* statistic [35] was negative in most cases both inside and outside the rearranged region (Table 2), indicating an excess of low frequency SNPs (single nucleotide polymorphisms) consistent with a population expansion in A. gambiae [36]. None of the values of Tajima's *D* were significant, under equilibrium population histories or more realistic scenarios with expanding populations. However, four values of the *R*_2_ statistic [37], also based on the site frequency spectrum, indicated a significant excess of low frequency polymorphisms relative to the neutral-equilibrium expectation. Also evident in measures of the site frequency spectrum—and consistent with the selective sweep hypothesis—were the more extreme values of both statistics in 2La-arrangement chromosomes. At seven of nine genes within the inversion, values of Tajima's *D* and *R*_2_ were lower (indicating a greater excess of low frequency polymorphisms) among 2La chromosomes. This result would be expected if the 2La arrangement rose in frequency quickly, though this explanation is somewhat undermined by the fact that Tajima's *D* and *R*_2_ are also lower at loci in collinear regions among individuals carrying the 2La arrangement.

### Selection Maintains 2La Polymorphism

Although no clear footprint of a recent selective sweep or of balancing selection was found in the nucleotide sequence data, it may still be the case that selection is responsible for the maintenance of the proximal and distal clusters in association with inversion 2La. Multiple SNPs in both the proximal cluster and the distal cluster are in perfect linkage disequilibrium (*D*′ = 1) with the inversion (i.e., they are fixed between inversion arrangements), even though they are quite distant from the breakpoints. The alternative to a selective explanation for their maintenance is that the observed linkage disequilibrium is an historical remnant of complete association dating from the time that the inversion entered the A. gambiae gene pool. Two lines of evidence suggest that this date is quite recent. First, A. gambiae itself is considered quite recently derived. Based on its strongly anthropophilic behavior and its dependence upon anthropogenic breeding sites, Coluzzi and colleagues [20] have argued that A. gambiae is the product of a speciation process originating in the central African rainforest and driven by human impact on the environment subsequent to the Neolithic revolution ∼10,000–12,000 years ago. Second, based on the assumption of a single introduction of 2La, we can derive an estimate for the age of 2La in A. gambiae that agrees fairly well with this time frame*.* After removing polymorphisms shared between arrangements and between species, we used nucleotide polymorphism data from the proximal breakpoint (i.e., *endp*) to estimate that the *E*[*T*_MRCA_] of our sample of 2La chromosomes is ∼2.7 *N_e_* generations (where N_e_ is the effective population size). Microsatellite-based estimates of *N_e_* of A. gambiae are reasonably consistent across Africa [38]. Values of *N_e_* obtained from Cameroon based on the infinite alleles or stepwise mutation models of mutation, respectively, were 11,500 and 49,000 [38]. This corresponds to the introduction of 2La into A. gambiae ∼3,000–11,000 years ago, assuming 12 generations per year.

Despite the relatively recent introduction of the 2La inversion into A. gambiae, we can distinguish between selective and neutral explanations for the maintenance of the inversion polymorphism by examining the amount of linkage disequilibrium expected between each of the clusters and their closest breakpoints given known rates of crossing-over. Using polymorphic microsatellite loci, Stump *et al* [30] estimated recombination rates on 2L from the backcross progeny of 2La/+^a^ heterokaryotypes and as a control, from 2L+^a^ homokaryotypes. They found that although recombination was at least 4× lower inside the inversion than in collinear regions, there were still appreciable levels of both gene conversion and crossing-over. From these data we estimate that the recombination fraction between the midpoint of the proximal cluster and the proximal breakpoint is *r* = 0.0012, and that the fraction between the midpoint of the distal cluster and the distal breakpoint is *r* = 0.0168. Given these estimates of recombination and *N_e_* = 11,500–49,000 [38], the quantity 4*N_e_r* is much greater than 1 for the regions in-between both clusters and their closest respective breakpoints. With 4*N_e_r* ≫ 1, the only linkage disequilibrium expected in a population should be due to sampling variance [39]; we find that the observed value of the non-normalized linkage disequilibrium coefficient, *D*, is highly significantly different than 0 between polymorphic sites in either cluster and the inversion (*p* = 1.08 × 10^−11^ for the smallest sample size of any locus in the proximal and distal clusters). This result strongly supports the conclusion that some form of natural selection must be maintaining the association between the individual clusters and the inversion, and therefore the inversion polymorphism itself.

As an alternative way of considering the highly unlikely nature of the values of linkage disequilibrium observed, recall that disequilibrium declines as *D_t_* = (1 − *r*)*^t^D*_0_, where *D_t_* is the disequilibrium expected after *t* generations starting from an initial value of *D*_0_. Given values of *r* (see above) and a starting value of *D*_0_ = 0.25 (i.e., complete linkage disequilibrium), we would expect *D_t_* to be less than 0.001 between the proximal cluster and the inversion in 4,600 generations and less than 0.001 between the distal cluster and the inversion in 190 generations. These numbers of generations translate to an almost complete lack of linkage disequilibrium after 380 years for the proximal cluster and 16 years for the distal cluster. If our estimates for the date of introduction of the 2La polymorphism are within even an order of magnitude of the correct time, these results suggest that more than enough time has elapsed for the decay of disequilibrium between these highly diverged regions and the inversion itself.

## Discussion

Use of the Affymetrix GeneChip microarray allowed us to map patterns of divergence in an inversion with unprecedented detail, leading to the discovery of two relatively small regions (the proximal and distal clusters) whose persistent association with the inversion is inconsistent with a neutral model. Below we discuss the forms of selection likely to be maintaining the inversion as a polymorphism in A. gambiae.

### Maintenance of 2La Polymorphism in A. gambiae

There have been numerous models proposed to explain the maintenance of inversion polymorphisms (reviewed in [2,40]). Perhaps the two most commonly cited are epistasis (coadaptation) among alleles within an inversion and overdominance of inversion heterokaryotypes [1]. Overdominance is an unlikely mechanism in this case. Multiple instances of stable geographic clines of 2La frequency along aridity gradients suggest that alternative arrangements are differentially adapted to dry and humid conditions, and that the cline results from a balance between migration and differential selection at opposite ends of an ecotone. This conclusion is reinforced by cyclical changes in 2La frequency associated with rainy and dry seasons each year. In addition, the overdominance hypothesis does not make clear predictions regarding linkage disequilibrium between loci within the inversion and the inversion itself, as a number of molecular mechanisms might be responsible for heterosis.

In contrast, Dobzhansky's coadapted gene hypothesis makes clear predictions about the nature of epistasis among alleles within the inverted region and linkage disequilibrium between these alleles and the inversion. The observation in *Drosophila* of linkage disequilibrium between inversions and genes only loosely linked to breakpoints has lead previous researchers to suggest that epistasis for fitness was maintaining these inversion polymorphisms, though epistasis was not directly evaluated in these studies [12,13,41].

An alternative selective hypothesis for the maintenance of inversions that does not require epistasis is also consistent with these findings. As in the case presented here, all of the inversion polymorphisms in which epistasis has been invoked exist along stable clines [12,13,41]. Population structure at multiple loci under selection across the cline can generate linkage disequilibrium among loci, in a multi-locus analog of the Wahlund effect [42]. This occurs because local adaptation to different environments at multiple loci can lead to parallel clines in allele frequencies, and therefore nonrandom associations among alleles. Under this model, migration-selection balance at two or more loci maintains the inversion; epistasis among the locally adapted alleles is not required and therefore the requirements of this model are less stringent than for coadaptation [8]. For A. gambiae, clines from arid to humid environments across Africa offer an ideal opportunity for local adaptation in multiple traits (see next section). Interbreeding among migrants carrying different genetic backgrounds on a collinear chromosome (e.g., humid- or arid-adapted) would create recombinants bearing fewer humid-adapted (arid-adapted) alleles, resulting in lower overall fitness under humid (arid) conditions. However, inversions that capture all humid-adapted alleles preserve their association in the face of immigrant arid-adapted genes (and vice versa). Thus, the inversion is maintained because it prevents recombination in the face of high levels of gene flow, as are observed in A. gambiae [43]. Further experiments will be needed to fully distinguish between the epistasis and local adaptation models for the maintenance of 2La.

### Targets of Selection

Our data predict that the proximal and distal clusters should contain at least some candidate genes that confer resistance to aridity on 2La (and tolerance to humidity on 2L+^a^), though it is important to emphasize that additional candidates can occur outside of these clusters and possibly outside of the inversion itself. Within their estimated boundaries, a total of 210 genes have been predicted in both clusters. The challenge of identifying candidate genes within clusters is complicated by the fact that in many cases there is little evidence supporting gene predictions, with poor or nonexistent functional annotation (Tables 1 and 2).

The effort is further complicated by an almost complete lack of information regarding the physiological and/or behavioral traits responsible for aridity tolerance conferred by 2La, which can include both desiccation resistance and resistance to heat stress. In the only published study of desiccation resistance and water balance in A. gambiae and A. arabiensis, a laboratory colony of A. arabiensis was significantly more resistant to desiccation than a colony of A. gambiae, due to higher initial body water content [44]. Metabolic rate, respiratory pattern, rate of water loss during desiccation, and water content at death were similar. As karyotype was not investigated nor controlled for during this study, these data are difficult to interpret with respect to the contribution of 2La; both colonies are known to be polymorphic for several inversions that have been associated with aridity in the field and the A. gambiae colony used was polymorphic for 2La. The same problem applies to the sole study of heat resistance that found A. arabiensis to be more heat tolerant than A. gambiae in a behavioral assay and stress test [45]. In the absence of more detailed guidance from empirical work, the most striking observation about gene content concerns the distal cluster, which contains the largest concentration of cuticle protein genes (40) in the A. gambiae genome, as well as three *hsp83* genes encoding heat shock proteins. However, the cuticle proteins are not present in the epicuticle, the layer primarily responsible for water retention [46]. Thus their role—if any—in heat or desiccation resistance remains obscure. Substantial additional effort will be required to pinpoint the important genes and to understand their contributions to adaptive phenotypes. As alluded to above, 2La is not the final story on resistance to desiccation; other inversions on 2R are also implicated in this trait [3,27]. Future progress will depend upon controlling for karyotype differences.

### Implications for Vectorial Capacity

In the group of sister species known as the A. gambiae complex, there is a clear correlation between inversion polymorphism and involvement in malaria transmission [3]. The least polymorphic species are relatively restricted in their geographic distributions and are only locally important vectors or—in two cases—non-vectors. On the other hand, A. arabiensis and A. gambiae are counted among the most important vectors of human malaria worldwide. They carry abundant inversion polymorphism and are distributed across most of tropical Africa and its diverse landscapes. The impact of inversion 2La on the distribution of A. gambiae has been particularly profound. Once acquired from A. arabiensis, it helped A. gambiae to spread outside of the humid rainforest into arid savannas. Polymorphism for this and other inversions has enabled an already proficient malaria vector to occupy a vastly expanded species range, consequently expanding malaria transmission. Our results have laid the groundwork for the functional genomics study of 2La which will illuminate not only the genetic basis of adaptations inside inversions, but also aspects of vector behavior relevant to control.

## Materials and Methods

### Mosquito collection, identification, and DNA isolation.

All mosquitoes used in this study were field-collected. Collections of A. gambiae and A. arabiensis were performed between May and September of 2005 in the village of Tibati, Cameroon (6°28′N, 12°37′E) by pyrethrum spray catch. *A. gambiae s.l.* were identified morphologically and the ovaries of half-gravid specimens dissected and fixed in Carnoy's solution (3:1 ethanol:glacial acetic acid). Sibling species and molecular forms M and S were identified using an rDNA assay [47]. Karyotyping was performed following standard protocol [48]. Inversion status of 2La was confirmed by a PCR diagnostic [49]. A. quadriannulatus specimens were collected in 1986 from southern Zimbabwe and kindly provided by F. Collins [50]. DNA was isolated from individual mosquitoes using the DNeasy Extraction Kit (Qiagen). The concentration of eluted DNA for each specimen was determined by spectrophotometry using the Nanodrop-1000 (Nanodrop Technologies).

### Microarray methods.

Fragmentation and labeling of 300 ng DNA from single specimens was achieved using random prime labeling in the presence of biotin-14-dCTP (BioPrime DNA Labeling System, Invitrogen) as described by J. Borevitz (http://naturalsystems.uchicago.edu/naturalvariation/methods/BorevitzSFPMethods.pdf). After purification by ethanol precipitation, labeled products were resuspended in 100 μl ddH_2_0. Quality and yield (estimated at ∼10 μg) were checked by electrophoresis of a 5 μl aliquot through a 2.5% agarose gel. Most products were ∼50 bp long. The remaining 95 μl of labeled genomic DNA was hybridized to the Affymetrix Anopheles/Plasmodium GeneChip using standard protocols for eukaryotic cRNA hybridization. Hybridization and scanning of arrays was performed by the Center for Medical Genomics, Indiana University Medical School. All arrays were processed under identical experimental conditions on the same day.

Cel files containing the raw probe intensity values were imported into Bioconductor (http://www.bioconductor.org), an open source software project based on the R programming language (http://www.r-project.org). Using the “affy” package, data quality was assessed to identify aberrant chips or spatial artifacts [51]. Approaches included examination of chip images of raw probe intensities at natural and log-scales, boxplot and histogram summaries of unprocessed log scale probe intensities for each array, and MA-plots. To visualize more subtle spatial artifacts, the affyPLM package was used to examine chip pseudo-images based on the probe level model (PLM) fit. Background adjustment and quantile normalization was performed using the Robust MultiArray Average (RMA) method without summarization by probeset [52]. Probe level data were exported as a comma separated value file for importation into Excel and are available from BJW upon request.

Probes from the Anopheles/Plasmodium GeneChip have been mapped against the A. gambiae reference genome (AgamP3). To identify any probes with exact matches to multiple genomic locations or secondary one-off mismatches, a list of all probes and their genomic locations was obtained through VectorBase (www.vectorbase.org) [53] from K. Megy. A Perl script (available from BJW upon request) was used to parse probes with exact matches to unique locations; those with multiple exact matches or additional single base pair mismatches were excluded from further analysis. For each of the 151,213 probes retained a two-tailed t-test was performed to compare background-adjusted and normalized hybridization intensity values obtained from the five 2La arrays versus the five 2L+^a^ arrays. Probes with *p*-values less than 0.01 were considered to contain SFPs between arrangements [14–16]. Overlapping significant probes were collapsed into one observation to control for nonindependence [16].

To test for overrepresentation of SFPs on 2L, we compared observed and expected numbers on 2L versus all other chromosomes combined, by a χ^2^ test. The expected number of SFPs in each category was calculated based on the genome-wide proportion of 0.89% as measured in this experiment. Similarly, overrepresentation of SFPs in the rearranged versus collinear part of 2L was tested by comparing observed and expected numbers given the 2L-specific proportion of 1.29%. An independent test of nonrandom SNP distribution on 2L that did not depend on prior information about the location of breakpoint sequences was implemented through a two-state HMM to identify differentiated versus homogenized regions along the arm. Transmission and emission probabilities of the HMM were estimated by expectation-maximization; hidden states were then inferred using the Viterbi algorithm in MATLAB (The MathWorks, http://www.mathworks.com/). To test for clustering of significant probes within the rearranged region, a sliding window analysis was performed with windows of 300 probes and a step-size of 20 probes. Each window was tested (χ^2^) for an excess of significant probes compared to the number expected by chance. A Bonferroni correction for multiple tests was conducted using the effective number of independent tests according to the relationship n* = *n*(1 − ρ)^2^, where *n* is the nominal number of tests conducted and ρ is the autocorrelation between successive test statistics [54,55].

### DNA sequence methods.

A. gambiae GeneBuild AgamP3.4 incorporates manual annotations of genes predicted on 2L. Based on the manual models, primers targeting exons were designed using Primer3 [56] and custom synthesized (Invitrogen). Primer sequences for each of the 11 exons studied and the corresponding VectorBase gene identifier is given in Table S1.

PCRs were carried out in a 50 μl reaction containing 200 μmol/l each dNTP, 2.5 mmol/l MgCl2, 2 mmol/l Tris-HCl (pH 8.4), 5 mmol/l KCl, 10 pmol of each primer, 5 U Taq polymerase, and ∼10 ng of template DNA. Thermocycler (MJ Research) conditions were 94 °C for 2 min; 35 cycles of 94 °C for 30 s, 58 °C for 30 s, 72 °C for 1 min; a final elongation at 72 °C for 10 min; and a 0 °C hold. All 50μl of the resulting products were separated on a 1.25% agarose gel stained with ethidium bromide. Products were excised and purified using the Geneclean Spin Kit (MP Biomedicals) or QIAquick Gel Extraction Kit (Qiagen).

PCR products were directly sequenced on both strands using an Applied Biosystems 3730xl DNA Analyzer and BigDye Terminator version 3.1 chemistry as recommended by the manufacturer. Electropherograms were trimmed and visually inspected for SNPs and heterozygous indels using Seqman II (DNASTAR, Madison, WI).

Haplotypes at each locus were reconstructed from the genotypic sequencing data using the PHASE (version 2.1) program, which implements a Bayesian statistical model for inferring haplotypes from population genotype data [57,58]. All default settings in PHASE were used except for tri-allelic and quad-allelic SNPs, for which the default assumption of stepwise mutation intended for microsatellite loci was relaxed. After two haplotypes were assigned to each specimen alignment was performed using ClustalX [59].

DnaSP version 4.10.9 was used to calculate standard polymorphism and divergence statistics and tests of neutrality [60]. Coalescent simulations of population expansion were conducted in ms [61], with populations exponentially growing starting at 2.7 N_e_ generations in the past. Significance of *F_ST_* values was based on 10,000 permutations conducted in Arlequin 3.11 [62]; significance of other values was determined from 10,000 coalescent simulations without recombination implemented in DnaSP [60]. A multilocus version of the HKA test of natural selection was implemented using HKA software developed and distributed by J. Hey (http://lifesci.rutgers.edu/~heylab/HeylabSoftware.htm#HKA). Using maximum composite likelihood distances [63], Neighbor-Joining gene trees were reconstructed in Mega4 [64].

To estimate the time to the most recent common ancestor of the 2La arrangement in A. gambiae, we used the expectation *E*[*T*_MRCA_] = 4*N_e_f*(1-*n*_i_^−1^), which is based on the number of segregating sites unique to each of the inverted and standard classes [65]*.* This estimate assumes that the 2La arrangement entered the population and instantaneously reached its current frequency. Violation of this assumption makes *E*[*T*_MRCA_] a minimum estimate of the age of the inverted class.

## Supporting Information

Table S1 Primer Pairs Used for Amplification and Sequencing of Genes Surveyed on A. gambiae Chromosome 2L (35 KB DOC)

Table S2 Predicted Genes in the Proximal Cluster (92 KB DOC)

Table S3 Predicted Genes in the Distal Cluster (436 KB DOC)

### Accession Numbers

All sequences mentioned in this paper have been deposited in the National Center for Biotechnology Information (NCBI) GenBank (http://www.ncbi.nlm.nih.gov/sites/gquery) under accession numbers EU097365 to EU097703.

## Abbreviations

*D*: linkage disequilibrium coefficient
*D*_*a*_: average net nucleotide divergence per site
HMM: hidden Markov model
Mb: megabase
*N_e_,*: effective population size
SFP: single feature polymorphism

## References

[pgen-0030217-b001] Dobzhansky T (1970). Genetics of the evolutionary process.

[pgen-0030217-b002] Krimbas CB, Powell JR, Krimbas CB, Powell JR (1992). Introduction. Drosophila inversion polymorphism.

[pgen-0030217-b003] Coluzzi M, Sabatini A, Petrarca V, Di Deco MA (1979). Chromosomal differentiation and adaptation to human environments in the Anopheles gambiae complex. Trans R Soc Trop Med Hyg.

[pgen-0030217-b004] Hoffmann AA, Sgro CM, Weeks AR (2004). Chromosomal inversion polymorphisms and adaptation. Trends Ecol Evol.

[pgen-0030217-b005] Stefansson H, Helgason A, Thorleifsson G, Steinthorsdottir V, Masson G (2005). A common inversion under selection in Europeans. Nat Genet.

[pgen-0030217-b006] Feder JL, Roethele JB, Filchak K, Niedbalski J, Romero-Severson J (2003). Evidence for inversion polymorphism related to sympatric host race formation in the apple maggot fly, Rhagoletis pomonella. Genetics.

[pgen-0030217-b007] Roberts PA, Ashburner M, Novitski E (1976). The genetics of chromosome aberration. The genetics and biology of Drosophila.

[pgen-0030217-b008] Kirkpatrick M, Barton N (2006). Chromosome inversions, local adaptation and speciation. Genetics.

[pgen-0030217-b009] Andolfatto P, Depaulis F, Navarro A (2001). Inversion polymorphisms and nucleotide variability in Drosophila. Genet Res.

[pgen-0030217-b010] Navarro A, Betran E, Barbadilla A, Ruiz A (1997). Recombination and gene flux caused by gene conversion and crossing over in inversion heterokaryotypes. Genetics.

[pgen-0030217-b011] Schaeffer SW, Anderson WW (2005). Mechanisms of genetic exchange within the chromosomal inversions of Drosophila pseudoobscura. Genetics.

[pgen-0030217-b012] Kennington WJ, Partridge L, Hoffmann AA (2006). Patterns of diversity and linkage disequilibrium within the cosmopolitan inversion In(3R)Payne in Drosophila melanogaster are indicative of coadaptation. Genetics.

[pgen-0030217-b013] Schaeffer SW, Goetting-Minesky MP, Kovacevic M, Peoples JR, Graybill JL (2003). Evolutionary genomics of inversions in Drosophila pseudoobscura: evidence for epistasis. Proc Natl Acad Sci U S A.

[pgen-0030217-b014] Borevitz JO, Liang D, Plouffe D, Chang HS, Zhu T (2003). Large-scale identification of single-feature polymorphisms in complex genomes. Genome Res.

[pgen-0030217-b015] Winzeler EA, Castillo-Davis CI, Oshiro G, Liang D, Richards DR (2003). Genetic diversity in yeast assessed with whole-genome oligonucleotide arrays. Genetics.

[pgen-0030217-b016] Turner TL, Hahn MW, Nuzhdin SV (2005). Genomic islands of speciation in Anopheles gambiae. PLoS Biol.

[pgen-0030217-b017] Rostoks N, Borevitz JO, Hedley PE, Russell J, Mudie S (2005). Single-feature polymorphism discovery in the barley transcriptome. Genome Biol.

[pgen-0030217-b018] Snow RW, Omumbo JA, Jamison DT, Feachem RG, Makgoba MW, Bos ER, Baingana FK (2006). Malaria. Disease and mortality in sub-Saharan Africa, 2nd edition.

[pgen-0030217-b019] Coluzzi M, Petrarca V, Di Deco MA (1985). Chromosomal inversion intergradation and incipient speciation in Anopheles gambiae. Boll Zool.

[pgen-0030217-b020] Coluzzi M, Sabatini A, Della Torre A, Di Deco MA, Petrarca V (2002). A polytene chromosome analysis of the Anopheles gambiae species complex. Science.

[pgen-0030217-b021] Besansky NJ, Krzywinski J, Lehmann T, Simard F, Kern M (2003). Semipermeable species boundaries between Anopheles gambiae and Anopheles arabiensis: evidence from multilocus DNA sequence variation. Proc Natl Acad Sci USA.

[pgen-0030217-b022] della Torre A, Merzagora L, Powell JR, Coluzzi M (1997). Selective introgression of paracentric inversions between two sibling species of the Anopheles gambiae complex. Genetics.

[pgen-0030217-b023] Coluzzi M (1992). Malaria vector analysis and control. Parasitology Today.

[pgen-0030217-b024] Bryan JH, Di Deco MA, Petrarca V, Coluzzi M (1982). Inversion polymorphism and incipient speciation in *Anopheles gambiae s. str.* in The Gambia, West Africa. Genetica.

[pgen-0030217-b025] Petrarca V, Sabatinelli G, Di Deco MA, Papakay M (1990). The Anopheles gambiae complex in the Federal Islamic Republic of Comoros (Indian Ocean): some cytogenetic and biometric data. Parassitologia.

[pgen-0030217-b026] Wondji C, Frederic S, Petrarca V, Etang J, Santolamazza F (2005). Species and populations of the Anopheles gambiae complex in Cameroon with special emphasis on chromosomal and molecular forms of *Anopheles gambiae s.s*. J Med Entomol.

[pgen-0030217-b027] Powell JR, Petrarca V, della Torre A, Caccone A, Coluzzi M (1999). Population structure, speciation, and introgression in the Anopheles gambiae complex. Parassitologia.

[pgen-0030217-b028] Holt RA, Subramanian GM, Halpern A, Sutton GG, Charlab R (2002). The genome sequence of the malaria mosquito Anopheles gambiae. Science.

[pgen-0030217-b029] Sharakhov IV, White BJ, Sharakhova MV, Kayondo J, Lobo NF (2006). Breakpoint structure reveals the unique origin of an interspecific chromosomal inversion (2La) in the Anopheles gambiae complex. Proc Natl Acad Sci U S A.

[pgen-0030217-b030] Stump AD, Pombi M, Goeddel L, Ribeiro JMC, Wilder JA (2007). Genetic exchange in 2La inversion heterokaryotypes of Anopheles gambiae. Insect Molecular Biology.

[pgen-0030217-b031] della Torre A, Tu Z, Petrarca V (2005). On the distribution and genetic differentiation of *Anopheles gambiae s.s.* molecular forms. Insect Biochem Mol Biol.

[pgen-0030217-b032] Clark AG (1997). Neutral behavior of shared polymorphism. Proc Natl Acad Sci U S A.

[pgen-0030217-b033] Ayala FJ, Coluzzi M (2005). Chromosome speciation: humans, Drosophila, and mosquitoes. Proc Natl Acad Sci U S A.

[pgen-0030217-b034] Hudson RR, Kreitman M, Aguade M (1987). A test of neutral molecular evolution based on nucleotide data. Genetics.

[pgen-0030217-b035] Tajima F (1989). Statistical method for testing the neutral mutation hypothesis by DNA polymorphism. Genetics.

[pgen-0030217-b036] Donnelly MJ, Licht MC, Lehmann T (2001). Evidence for recent population expansion in the evolutionary history of the malaria vectors Anopheles arabiensis and Anopheles gambiae. Molecular Biology and Evolution.

[pgen-0030217-b037] Ramos-Onsins SE, Rozas J (2002). Statistical properties of new neutrality tests against population growth. Mol Biol Evol.

[pgen-0030217-b038] Pinto J, Donnelly MJ, Sousa CA, Malta-Vacas J, Gil V (2003). An island within an island: genetic differentiation of Anopheles gambiae in Sao Tome, West Africa, and its relevance to malaria vector control. Heredity.

[pgen-0030217-b039] Hill WG, Robertson A (1968). Linkage disequilibrium in finite populations. Theor Appl Genet.

[pgen-0030217-b040] Powell JR (1997). Progress and prospects in evolutionary biology: the *Drosophila* model.

[pgen-0030217-b041] Van Delden W, Kamping A (1989). The association between the polymorphisms at the Adh and αGpdh loci and the In(2L)t inversion in Drosophila melanogaster in relation to temperature. Evolution.

[pgen-0030217-b042] Li W-H, Nei M (1974). Stable linkage disequilibrium without epistasis in subdivided populations. Theor Popul Biol.

[pgen-0030217-b043] Lehmann T, Licht M, Elissa N, Maega BT, Chimumbwa JM (2003). Population Structure of Anopheles gambiae in Africa. J Hered.

[pgen-0030217-b044] Gray EM, Bradley TJ (2005). Physiology of desiccation resistance in Anopheles gambiae and Anopheles arabiensis. Am J Trop Med Hyg.

[pgen-0030217-b045] Kirby MJ, Lindsay SW (2004). Responses of adult mosquitoes of two sibling species, Anopheles arabiensis and *A. gambiae s.s*. (Diptera: Culicidae), to high temperatures. Bull Entomol Res.

[pgen-0030217-b046] Willis JH, Iconomidou VA, Smith RF, Hamodrakas SJ, Gilbert LI, Iatrou K, Gill SS (2005). Cuticular proteins. Comprehensive molecular insect science.

[pgen-0030217-b047] Fanello C, Santolamazza F, della Torre A (2002). Simultaneous identification of species and molecular forms of the Anopheles gambiae complex by PCR-RFLP. Med Vet Entomol.

[pgen-0030217-b048] della Torre A, Crampton JM, Beard CB, Louis C (1997). Polytene chromosome preparation from anopheline mosquitoes. Molecular biology of disease vectors: a methods manual.

[pgen-0030217-b049] White BJ, Santolamazza F, Kamau L, Pombi M, Grushko O (2007). Molecular karyotyping of the 2La inversion in Anopheles gambiae. Am J Trop Med Hyg.

[pgen-0030217-b050] Collins FH, Petrarca V, Mpofu S, Brandling-Bennett AD, Were JB (1988). Comparison of DNA probe and cytogenetic methods for identifying field collected Anopheles gambiae complex mosquitoes. Am J Trop Med Hyg.

[pgen-0030217-b051] Bolstad BM, Irizarry RA, Gautier L, Wu Z, Gentleman R, Carey V, Huber W, Irizarry R, Dudoit S (2005). Preprocessing high-density oligonucleotide arrays. Bioinformatics and computational biology solutions using R and Bioconductor.

[pgen-0030217-b052] Irizarry RA, Hobbs B, Collin F, Beazer-Barclay YD, Antonellis KJ (2003). Exploration, normalization, and summaries of high density oligonucleotide array probe level data. Biostatistics.

[pgen-0030217-b053] Lawson D, Arensburger P, Atkinson P, Besansky NJ, Bruggner RV (2007). VectorBase: a home for invertebrate vectors of human pathogens. Nucleic Acids Res Vol.

[pgen-0030217-b054] Dawdy DR, Matalas NC, Te Chow V (1964). Analysis of variance, covariance, and time series: Chap. 8-III. Handbook of applied hydrology.

[pgen-0030217-b055] Hahn MW (2006). Accurate inference and estimation in population genomics. Mol Biol Evol.

[pgen-0030217-b056] Rozen S, Skaletsky HJ, Krawetz S, Misener S (2000). Primer3 on the WWW for general users and for biologist programmers. Bioinformatics methods and protocols: methods in molecular biology.

[pgen-0030217-b057] Stephens M, Donnelly P (2003). A comparison of bayesian methods for haplotype reconstruction from population genotype data. Am J Hum Genet.

[pgen-0030217-b058] Stephens M, Smith NJ, Donnelly P (2001). A new statistical method for haplotype reconstruction from population data. Am J Hum Genet.

[pgen-0030217-b059] Thompson JD, Gibson TJ, Plewniak F, Jeanmougin F, Higgins DG (1997). The ClustalX windows interface: flexible strategies for multiple sequence alignment aided by quality analysis tools. Nucleic Acids Res.

[pgen-0030217-b060] Rozas J, Sanchez-DelBarrio JC, Messeguer X, Rozas R (2003). DnaSP, DNA polymorphism analyses by the coalescent and other methods. Bioinformatics.

[pgen-0030217-b061] Hudson RR (2002). Generating samples under a Wright-Fisher neutral model of genetic variation. Bioinformatics.

[pgen-0030217-b062] Excoffier L, Laval G, Schneider S (2005). Arlequin ver. 3.0: an integrated software package for population genetics data analysis. Evolutionary Bioinformatics Online.

[pgen-0030217-b063] Tamura K, Nei M, Kumar S (2004). Prospects for inferring very large phylogenies by using the neighbor-joining method. Proc Natl Acad Sci U S A.

[pgen-0030217-b064] Tamura K, Dudley J, Nei M, Kumar S (2007). MEGA4: molecular evolutionary genetics analysis (MEGA) software version 4.0. Mol Biol Evol.

[pgen-0030217-b065] Andolfatto P, Wall JD, Kreitman M (1999). Unusual haplotype structure at the proximal breakpoint of In(2L)t in a natural population of Drosophila melanogaster. Genetics.

[pgen-0030217-b066] Nei M (1987). Molecular Evolutionary Genetics.

[pgen-0030217-b067] Hudson RR, Slatkin M, Maddison WP (1992). Estimation of levels of gene flow from DNA sequence data. Genetics.

