# Supplementary material for: Localization of Candidate Regions Maintaining a Common Polymorphic Inversion (2La) in Anopheles gambiae
Source: PLoS Genet. 2007 Dec 7;3(12):e217. doi: 10.1371/journal.pgen.0030217 (PMC2134946; doi:10.1371/journal.pgen.0030217)
Supplement: Supplementary file 1 [file pgen.0030217.st001.doc]

Supplementary Table S1. Primer pairs used for amplification and sequencing of genes

surveyed on *A. gambiae* chromosome 2L.

| Locus | VectorBase Identifier | Primer Sequences (5’-3’) | AgamP3 Positions |
| --- | --- | --- | --- |
| *lys-c* | AGAP005717 | F: CAAACGGTGTGCAGTACCAC  R: CTTCATGCACTCCACGTCAT | 19563746-19563765  19563161-19563180 |
| *endp* | AGAP005792 | F: AAGCTAAGCGCCTCCAGATA  R: GCTCAGAAAACGCAAACACA | 20721840-20721859  20721254-20721273 |
| *unk* | AGAP005819 | F: CTCTTTGAGCCGAACGAAAC  R: AATCATCCACCAGCGTTAGC | 21834046-21834065  21834783-21834802 |
| *asph* | AGAP005832 | F: TAAAGCCGTACTGCACGAGA  R: GGCCACCCGTATGTAGGTC | 22294333-22294352  22295014-22295032 |
| *hdac* | AGAP006511 | F: TCGGAAATCTGCATCAACTG  R: ACTCGGTCGTGTTCTGGTTC | 33290106-33290125  33290774-33290793 |
| *nwk* | AGAP006774 | F: GCAATTTCCGACTTGCGTAT  R: GAAGGGCTCCTTCTTCCAGT | 38119880-38119899  38120499-38120518 |
| *cpr34* | AGAP006884 | F: CACTGACTGTCGCCTGTTTG  R: CATAGCGCCGATTCTGATTT | 39221719-39221738  39222135-39222154 |
| *cpr63* | AGAP006866 | F: TCCAGACCAAACACTCATCG  R: AACCTTCCGCTTCATCGTAG | 39227185-39227204  39227600-39227619 |
| *srpn2* | AGAP006911 | F: CGGTGAAAATTCTGCTCACA  R: CACCTCGTTCTCCTCCATGT | 39898460-39898479  39899144-39899163 |
| *depcd5* | AGAP007010 | F: TAACGATCGTGCTGTTCTCG  R: TCGGTACGGACGTTTTCTTC | 40901645-40901664  40902486-40902505 |
| *znf294* | AGAP007143 | F: CATTATCGAATGTGCGGTTG  R: TCGCGACTGTTTCGTTATCA | 43570656-43570670  43569988-43570007 |
