# Supplementary material for: Localization of Candidate Regions Maintaining a Common Polymorphic Inversion (2La) in Anopheles gambiae
Source: PLoS Genet. 2007 Dec 7;3(12):e217. doi: 10.1371/journal.pgen.0030217 (PMC2134946; doi:10.1371/journal.pgen.0030217)
Supplement: Supplementary file 2 [file pgen.0030217.st002.doc]

Supplementary Table S2. Predicted genes in proximal cluster 1

| **Ensembl Gene ID** | **Description^1^** | **Gene Start (bp)** | **Gene End (bp)** | **Affy Probe ID** |
| --- | --- | --- | --- | --- |
| [AGAP005799](http://www.ensembl.org/Anopheles_gambiae/geneview?gene=AGAP005799) | cell cycle regulation; signaling | [21132976](http://www.ensembl.org/Anopheles_gambiae/contigview?chr=2L&vc_start=21132976&vc_end=21163656) | [21163656](http://www.ensembl.org/Anopheles_gambiae/contigview?chr=2L&vc_start=21132976&vc_end=21163656) | Ag.2L.1385.0_CDS_at, Ag.2L.1973.0_CDS_at |
| [AGAP005800](http://www.ensembl.org/Anopheles_gambiae/geneview?gene=AGAP005800) | chromatin binding; DNA replication | [21192433](http://www.ensembl.org/Anopheles_gambiae/contigview?chr=2L&vc_start=21192433&vc_end=21194835) | [21194835](http://www.ensembl.org/Anopheles_gambiae/contigview?chr=2L&vc_start=21192433&vc_end=21194835) | Ag.2L.612.0_CDS_at |
| [AGAP005801](http://www.ensembl.org/Anopheles_gambiae/geneview?gene=AGAP005801) | Rho GTPase activator | [21195428](http://www.ensembl.org/Anopheles_gambiae/contigview?chr=2L&vc_start=21195428&vc_end=21197032) | [21197032](http://www.ensembl.org/Anopheles_gambiae/contigview?chr=2L&vc_start=21195428&vc_end=21197032) |  |
| [AGAP005802](http://www.ensembl.org/Anopheles_gambiae/geneview?gene=AGAP005802) | 60S ribosomal protein L8 | [21199038](http://www.ensembl.org/Anopheles_gambiae/contigview?chr=2L&vc_start=21199038&vc_end=21200529) | [21200529](http://www.ensembl.org/Anopheles_gambiae/contigview?chr=2L&vc_start=21199038&vc_end=21200529) | Ag.2L.2.0_CDS_s_at |
| [AGAP005803](http://www.ensembl.org/Anopheles_gambiae/geneview?gene=AGAP005803) |  | [21202719](http://www.ensembl.org/Anopheles_gambiae/contigview?chr=2L&vc_start=21202719&vc_end=21225559) | [21225559](http://www.ensembl.org/Anopheles_gambiae/contigview?chr=2L&vc_start=21202719&vc_end=21225559) | Ag.2L.2486.0_CDS_at, Ag.2L.1631.0_CDS_at, Ag.2L.1197.0_CDS_at |
| [AGAP005804](http://www.ensembl.org/Anopheles_gambiae/geneview?gene=AGAP005804) | forkhead box; DNA binding; transcriptional regulation | [21233301](http://www.ensembl.org/Anopheles_gambiae/contigview?chr=2L&vc_start=21233301&vc_end=21234686) | [21234686](http://www.ensembl.org/Anopheles_gambiae/contigview?chr=2L&vc_start=21233301&vc_end=21234686) | Ag.2L.1975.0_CDS_at |
| [AGAP005805](http://www.ensembl.org/Anopheles_gambiae/geneview?gene=AGAP005805) | signal transduction | [21358697](http://www.ensembl.org/Anopheles_gambiae/contigview?chr=2L&vc_start=21358697&vc_end=21360461) | [21360461](http://www.ensembl.org/Anopheles_gambiae/contigview?chr=2L&vc_start=21358697&vc_end=21360461) | Ag.2L.2488.0_CDS_at |
| [AGAP005806](http://www.ensembl.org/Anopheles_gambiae/geneview?gene=AGAP005806) | sphingomyelin phosphodiesterase | [21399276](http://www.ensembl.org/Anopheles_gambiae/contigview?chr=2L&vc_start=21399276&vc_end=21483674) | [21483674](http://www.ensembl.org/Anopheles_gambiae/contigview?chr=2L&vc_start=21399276&vc_end=21483674) | Ag.2L.1977.0_CDS_at, Ag.2L.1976.0_CDS_a_at, Ag.2L.1978.0_CDS_at |
| [AGAP005807](http://www.ensembl.org/Anopheles_gambiae/geneview?gene=AGAP005807) | inositol or phosphatidylinositol phosphatase | [21508765](http://www.ensembl.org/Anopheles_gambiae/contigview?chr=2L&vc_start=21508765&vc_end=21610766) | [21610766](http://www.ensembl.org/Anopheles_gambiae/contigview?chr=2L&vc_start=21508765&vc_end=21610766) |  |
| [AGAP005808](http://www.ensembl.org/Anopheles_gambiae/geneview?gene=AGAP005808) |  | [21542045](http://www.ensembl.org/Anopheles_gambiae/contigview?chr=2L&vc_start=21542045&vc_end=21545393) | [21545393](http://www.ensembl.org/Anopheles_gambiae/contigview?chr=2L&vc_start=21542045&vc_end=21545393) | Ag.2L.1980.0_CDS_at |
| [AGAP005809](http://www.ensembl.org/Anopheles_gambiae/geneview?gene=AGAP005809) | phosphatase inhibitor | [21620403](http://www.ensembl.org/Anopheles_gambiae/contigview?chr=2L&vc_start=21620403&vc_end=21623334) | [21623334](http://www.ensembl.org/Anopheles_gambiae/contigview?chr=2L&vc_start=21620403&vc_end=21623334) | Ag.2L.1633.0_CDS_a_at |
| [AGAP005810](http://www.ensembl.org/Anopheles_gambiae/geneview?gene=AGAP005810) | xylosyltransferase II | [21624117](http://www.ensembl.org/Anopheles_gambiae/contigview?chr=2L&vc_start=21624117&vc_end=21626855) | [21626855](http://www.ensembl.org/Anopheles_gambiae/contigview?chr=2L&vc_start=21624117&vc_end=21626855) |  |
| [AGAP005811](http://www.ensembl.org/Anopheles_gambiae/geneview?gene=AGAP005811) | xylosyltransferase II | [21627556](http://www.ensembl.org/Anopheles_gambiae/contigview?chr=2L&vc_start=21627556&vc_end=21630573) | [21630573](http://www.ensembl.org/Anopheles_gambiae/contigview?chr=2L&vc_start=21627556&vc_end=21630573) | Ag.2L.2489.0_CDS_at, Ag.2L.1981.0_CDS_a_at |
| [AGAP005812](http://www.ensembl.org/Anopheles_gambiae/geneview?gene=AGAP005812) | RNA polymerase II transcription elongation factor | [21631168](http://www.ensembl.org/Anopheles_gambiae/contigview?chr=2L&vc_start=21631168&vc_end=21632196) | [21632196](http://www.ensembl.org/Anopheles_gambiae/contigview?chr=2L&vc_start=21631168&vc_end=21632196) | Ag.2L.2489.0_CDS_at, Ag.2L.1981.0_CDS_a_at |
| [AGAP005813](http://www.ensembl.org/Anopheles_gambiae/geneview?gene=AGAP005813) | NifU-like; iron ion binding | [21632619](http://www.ensembl.org/Anopheles_gambiae/contigview?chr=2L&vc_start=21632619&vc_end=21633346) | [21633346](http://www.ensembl.org/Anopheles_gambiae/contigview?chr=2L&vc_start=21632619&vc_end=21633346) | Ag.2L.263.0_CDS_at |
| [AGAP005814](http://www.ensembl.org/Anopheles_gambiae/geneview?gene=AGAP005814) | ecdysoneless homolog | [21634351](http://www.ensembl.org/Anopheles_gambiae/contigview?chr=2L&vc_start=21634351&vc_end=21636655) | [21636655](http://www.ensembl.org/Anopheles_gambiae/contigview?chr=2L&vc_start=21634351&vc_end=21636655) | Ag.2L.1386.0_CDS_a_at |
| [AGAP005815](http://www.ensembl.org/Anopheles_gambiae/geneview?gene=AGAP005815) | YLP motif, possible substrate for tyrosine kinase | [21638346](http://www.ensembl.org/Anopheles_gambiae/contigview?chr=2L&vc_start=21638346&vc_end=21640590) | [21640590](http://www.ensembl.org/Anopheles_gambiae/contigview?chr=2L&vc_start=21638346&vc_end=21640590) | Ag.2L.1198.0_CDS_at, Ag.2L.1386.0_CDS_a_at |
| [AGAP005816](http://www.ensembl.org/Anopheles_gambiae/geneview?gene=AGAP005816) | neurotransmitter secretion | [21761237](http://www.ensembl.org/Anopheles_gambiae/contigview?chr=2L&vc_start=21761237&vc_end=21821057) | [21821057](http://www.ensembl.org/Anopheles_gambiae/contigview?chr=2L&vc_start=21761237&vc_end=21821057) | Ag.2L.1983.0_CDS_at |
| [AGAP005817](http://www.ensembl.org/Anopheles_gambiae/geneview?gene=AGAP005817) | cyclin-dependent protein kinase | [21826613](http://www.ensembl.org/Anopheles_gambiae/contigview?chr=2L&vc_start=21826613&vc_end=21829719) | [21829719](http://www.ensembl.org/Anopheles_gambiae/contigview?chr=2L&vc_start=21826613&vc_end=21829719) | Ag.2L.1199.0_CDS_at |
| [AGAP005818](http://www.ensembl.org/Anopheles_gambiae/geneview?gene=AGAP005818) | protein binding | [21831271](http://www.ensembl.org/Anopheles_gambiae/contigview?chr=2L&vc_start=21831271&vc_end=21832371) | [21832371](http://www.ensembl.org/Anopheles_gambiae/contigview?chr=2L&vc_start=21831271&vc_end=21832371) | Ag.2L.886.0_CDS_at |
| [AGAP005819](http://www.ensembl.org/Anopheles_gambiae/geneview?gene=AGAP005819) | chromatin binding; DNA replication | [21832968](http://www.ensembl.org/Anopheles_gambiae/contigview?chr=2L&vc_start=21832968&vc_end=21835239) | [21835239](http://www.ensembl.org/Anopheles_gambiae/contigview?chr=2L&vc_start=21832968&vc_end=21835239) | Ag.2L.1200.0_CDS_at, Ag.2L.1387.0_CDS_at |
| [AGAP005820](http://www.ensembl.org/Anopheles_gambiae/geneview?gene=AGAP005820) | protein binding | [21835318](http://www.ensembl.org/Anopheles_gambiae/contigview?chr=2L&vc_start=21835318&vc_end=21836802) | [21836802](http://www.ensembl.org/Anopheles_gambiae/contigview?chr=2L&vc_start=21835318&vc_end=21836802) | Ag.2L.1200.0_CDS_at, Ag.2L.1387.0_CDS_at |
| [AGAP005821](http://www.ensembl.org/Anopheles_gambiae/geneview?gene=AGAP005821) | miRNA aga-mir-219 | [21839671](http://www.ensembl.org/Anopheles_gambiae/contigview?chr=2L&vc_start=21839671&vc_end=21839758) | [21839758](http://www.ensembl.org/Anopheles_gambiae/contigview?chr=2L&vc_start=21839671&vc_end=21839758) |  |
| [AGAP005822](http://www.ensembl.org/Anopheles_gambiae/geneview?gene=AGAP005822) |  | [21865434](http://www.ensembl.org/Anopheles_gambiae/contigview?chr=2L&vc_start=21865434&vc_end=21867120) | [21867120](http://www.ensembl.org/Anopheles_gambiae/contigview?chr=2L&vc_start=21865434&vc_end=21867120) | Ag.2L.1077.0_CDS_at |
| [AGAP005823](http://www.ensembl.org/Anopheles_gambiae/geneview?gene=AGAP005823) | ubiquitin-like protein | [22049966](http://www.ensembl.org/Anopheles_gambiae/contigview?chr=2L&vc_start=22049966&vc_end=22051521) | [22051521](http://www.ensembl.org/Anopheles_gambiae/contigview?chr=2L&vc_start=22049966&vc_end=22051521) | Ag.2L.494.0_CDS_at |
| [AGAP005824](http://www.ensembl.org/Anopheles_gambiae/geneview?gene=AGAP005824) |  | [22051819](http://www.ensembl.org/Anopheles_gambiae/contigview?chr=2L&vc_start=22051819&vc_end=22052908) | [22052908](http://www.ensembl.org/Anopheles_gambiae/contigview?chr=2L&vc_start=22051819&vc_end=22052908) | Ag.2L.3843.0_at, Ag.2L.339.0_CDS_a_at, Ag.2L.981.0_CDS_at |
| [AGAP005825](http://www.ensembl.org/Anopheles_gambiae/geneview?gene=AGAP005825) | lipid kinase | [22052936](http://www.ensembl.org/Anopheles_gambiae/contigview?chr=2L&vc_start=22052936&vc_end=22054542) | [22054542](http://www.ensembl.org/Anopheles_gambiae/contigview?chr=2L&vc_start=22052936&vc_end=22054542) | Ag.2L.3843.0_at, Ag.2L.339.0_CDS_a_at, Ag.2L.981.0_CDS_at |
| [AGAP005826](http://www.ensembl.org/Anopheles_gambiae/geneview?gene=AGAP005826) | SH2 domain binding | [22057270](http://www.ensembl.org/Anopheles_gambiae/contigview?chr=2L&vc_start=22057270&vc_end=22066474) | [22066474](http://www.ensembl.org/Anopheles_gambiae/contigview?chr=2L&vc_start=22057270&vc_end=22066474) | Ag.2L.1388.0_CDS_at |
| [AGAP005827](http://www.ensembl.org/Anopheles_gambiae/geneview?gene=AGAP005827) | tRNA-Tyr | [22061211](http://www.ensembl.org/Anopheles_gambiae/contigview?chr=2L&vc_start=22061211&vc_end=22061311) | [22061311](http://www.ensembl.org/Anopheles_gambiae/contigview?chr=2L&vc_start=22061211&vc_end=22061311) | Ag.2L.1388.0_CDS_at |
| [AGAP005828](http://www.ensembl.org/Anopheles_gambiae/geneview?gene=AGAP005828) |  | [22068864](http://www.ensembl.org/Anopheles_gambiae/contigview?chr=2L&vc_start=22068864&vc_end=22086035) | [22086035](http://www.ensembl.org/Anopheles_gambiae/contigview?chr=2L&vc_start=22068864&vc_end=22086035) | Ag.2L.613.1_a_at, Ag.2L.613.0_CDS_at |
| [AGAP005829](http://www.ensembl.org/Anopheles_gambiae/geneview?gene=AGAP005829) | microtubule-associated complex | [22088475](http://www.ensembl.org/Anopheles_gambiae/contigview?chr=2L&vc_start=22088475&vc_end=22089755) | [22089755](http://www.ensembl.org/Anopheles_gambiae/contigview?chr=2L&vc_start=22088475&vc_end=22089755) | Ag.2L.646.0_CDS_a_at |
| [AGAP005830](http://www.ensembl.org/Anopheles_gambiae/geneview?gene=AGAP005830) | transcription factor | [22091909](http://www.ensembl.org/Anopheles_gambiae/contigview?chr=2L&vc_start=22091909&vc_end=22094208) | [22094208](http://www.ensembl.org/Anopheles_gambiae/contigview?chr=2L&vc_start=22091909&vc_end=22094208) | Ag.2L.2490.0_CDS_at |

^1^Based on annotation from AgamP3.4 if available, or AnoXcel ver. Feb 2006 (www.vectorbase.org).
