# Supplementary material for: Localization of Candidate Regions Maintaining a Common Polymorphic Inversion (2La) in Anopheles gambiae
Source: PLoS Genet. 2007 Dec 7;3(12):e217. doi: 10.1371/journal.pgen.0030217 (PMC2134946; doi:10.1371/journal.pgen.0030217)
Supplement: Supplementary file 3 [file pgen.0030217.st003.doc]

Supplementary Table S3. Predicted genes in distal cluster 2.

| **Ensembl Gene ID** | **Description**^1^ | **Gene Start (bp)** | **Gene End (bp)** | **Affy Probe ID** |
| --- | --- | --- | --- | --- |
| [AGAP006814](http://www.ensembl.org/Anopheles_gambiae/geneview?gene=AGAP006814) | transcription regulation | [38856915](http://www.ensembl.org/Anopheles_gambiae/contigview?chr=2L&vc_start=38856915&vc_end=38858249) | [38858249](http://www.ensembl.org/Anopheles_gambiae/contigview?chr=2L&vc_start=38856915&vc_end=38858249) | Ag.2L.829.0_CDS_at |
| [AGAP006815](http://www.ensembl.org/Anopheles_gambiae/geneview?gene=AGAP006815) | supervillin-like; actin binding | [38858563](http://www.ensembl.org/Anopheles_gambiae/contigview?chr=2L&vc_start=38858563&vc_end=38859832) | [38859832](http://www.ensembl.org/Anopheles_gambiae/contigview?chr=2L&vc_start=38858563&vc_end=38859832) | Ag.2L.376.0_CDS_at, Ag.2L.289.1_a_at |
| [AGAP006816](http://www.ensembl.org/Anopheles_gambiae/geneview?gene=AGAP006816) |  | [38859814](http://www.ensembl.org/Anopheles_gambiae/contigview?chr=2L&vc_start=38859814&vc_end=38861198) | [38861198](http://www.ensembl.org/Anopheles_gambiae/contigview?chr=2L&vc_start=38859814&vc_end=38861198) | Ag.2L.376.0_CDS_at, Ag.2L.289.1_a_at |
| [AGAP006817](http://www.ensembl.org/Anopheles_gambiae/geneview?gene=AGAP006817) | regulation of chromatin (dis)assembly | [38862220](http://www.ensembl.org/Anopheles_gambiae/contigview?chr=2L&vc_start=38862220&vc_end=38867043) | [38867043](http://www.ensembl.org/Anopheles_gambiae/contigview?chr=2L&vc_start=38862220&vc_end=38867043) | Ag.2L.830.0_CDS_at, Ag.2L.376.0_CDS_at |
| [AGAP006818](http://www.ensembl.org/Anopheles_gambiae/geneview?gene=AGAP006818) | ribonucleoside-diphosphate reductase; DNA replicaton | [38869110](http://www.ensembl.org/Anopheles_gambiae/contigview?chr=2L&vc_start=38869110&vc_end=38873116) | [38873116](http://www.ensembl.org/Anopheles_gambiae/contigview?chr=2L&vc_start=38869110&vc_end=38873116) | Ag.2L.297.0_CDS_a_at, Ag.2L.297.0_UTR_a_at |
| [AGAP006819](http://www.ensembl.org/Anopheles_gambiae/geneview?gene=AGAP006819) |  | [38882380](http://www.ensembl.org/Anopheles_gambiae/contigview?chr=2L&vc_start=38882380&vc_end=38884506) | [38884506](http://www.ensembl.org/Anopheles_gambiae/contigview?chr=2L&vc_start=38882380&vc_end=38884506) | Ag.2L.4010.0_at, Ag.2L.916.1_CDS_a_at |
| [AGAP006820](http://www.ensembl.org/Anopheles_gambiae/geneview?gene=AGAP006820) | peroxisome-targeting signal receptor | [38887650](http://www.ensembl.org/Anopheles_gambiae/contigview?chr=2L&vc_start=38887650&vc_end=38890117) | [38890117](http://www.ensembl.org/Anopheles_gambiae/contigview?chr=2L&vc_start=38887650&vc_end=38890117) | Ag.2L.1455.0_CDS_at, Ag.2L.195.1_CDS_s_at |
| [AGAP006821](http://www.ensembl.org/Anopheles_gambiae/geneview?gene=AGAP006821) | acetyl CoA C-acetyltransferase | [38890935](http://www.ensembl.org/Anopheles_gambiae/contigview?chr=2L&vc_start=38890935&vc_end=38893474) | [38893474](http://www.ensembl.org/Anopheles_gambiae/contigview?chr=2L&vc_start=38890935&vc_end=38893474) | Ag.2L.195.1_CDS_s_at, Ag.2L.195.0_CDS_s_at |
| [AGAP006822](http://www.ensembl.org/Anopheles_gambiae/geneview?gene=AGAP006822) |  | [38895037](http://www.ensembl.org/Anopheles_gambiae/contigview?chr=2L&vc_start=38895037&vc_end=38907809) | [38907809](http://www.ensembl.org/Anopheles_gambiae/contigview?chr=2L&vc_start=38895037&vc_end=38907809) |  |
| [AGAP006823](http://www.ensembl.org/Anopheles_gambiae/geneview?gene=AGAP006823) |  | [38915890](http://www.ensembl.org/Anopheles_gambiae/contigview?chr=2L&vc_start=38915890&vc_end=38951679) | [38951679](http://www.ensembl.org/Anopheles_gambiae/contigview?chr=2L&vc_start=38915890&vc_end=38951679) | Ag.2L.917.0_CDS_at |
| [AGAP006824](http://www.ensembl.org/Anopheles_gambiae/geneview?gene=AGAP006824) | 5-formyltetrahydrofolate cyclo-ligase | [38955405](http://www.ensembl.org/Anopheles_gambiae/contigview?chr=2L&vc_start=38955405&vc_end=38962892) | [38962892](http://www.ensembl.org/Anopheles_gambiae/contigview?chr=2L&vc_start=38955405&vc_end=38962892) | Ag.2L.2668.1_a_at |
| [AGAP006825](http://www.ensembl.org/Anopheles_gambiae/geneview?gene=AGAP006825) | protein kinase | [38996806](http://www.ensembl.org/Anopheles_gambiae/contigview?chr=2L&vc_start=38996806&vc_end=39030002) | [39030002](http://www.ensembl.org/Anopheles_gambiae/contigview?chr=2L&vc_start=38996806&vc_end=39030002) | Ag.2L.2125.0_CDS_at, Ag.2L.2126.0_CDS_at |
| [AGAP006826](http://www.ensembl.org/Anopheles_gambiae/geneview?gene=AGAP006826) | integrin alpha PS3 receptor; possible laminin receptor | [39032112](http://www.ensembl.org/Anopheles_gambiae/contigview?chr=2L&vc_start=39032112&vc_end=39036699) | [39036699](http://www.ensembl.org/Anopheles_gambiae/contigview?chr=2L&vc_start=39032112&vc_end=39036699) | Ag.2L.1713.0_CDS_at |
| [AGAP006827](http://www.ensembl.org/Anopheles_gambiae/geneview?gene=AGAP006827) |  | [39037404](http://www.ensembl.org/Anopheles_gambiae/contigview?chr=2L&vc_start=39037404&vc_end=39038416) | [39038416](http://www.ensembl.org/Anopheles_gambiae/contigview?chr=2L&vc_start=39037404&vc_end=39038416) | Ag.2L.831.0_CDS_at |
| [AGAP006828](http://www.ensembl.org/Anopheles_gambiae/geneview?gene=AGAP006828) | Cuticular protein 60; RR-2 family | [39050620](http://www.ensembl.org/Anopheles_gambiae/contigview?chr=2L&vc_start=39050620&vc_end=39053268) | [39053268](http://www.ensembl.org/Anopheles_gambiae/contigview?chr=2L&vc_start=39050620&vc_end=39053268) | Ag.2L.1115.0_CDS_a_at |
| [AGAP006829](http://www.ensembl.org/Anopheles_gambiae/geneview?gene=AGAP006829) | Cuticular protein 59; RR-2 family | [39074233](http://www.ensembl.org/Anopheles_gambiae/contigview?chr=2L&vc_start=39074233&vc_end=39077008) | [39077008](http://www.ensembl.org/Anopheles_gambiae/contigview?chr=2L&vc_start=39074233&vc_end=39077008) | Ag.2L.918.0_UTR_a_at, Ag.2L.918.0_CDS_a_at |
| [AGAP006830](http://www.ensembl.org/Anopheles_gambiae/geneview?gene=AGAP006830) | Cuticular protein 58; RR-2 family | [39087872](http://www.ensembl.org/Anopheles_gambiae/contigview?chr=2L&vc_start=39087872&vc_end=39093776) | [39093776](http://www.ensembl.org/Anopheles_gambiae/contigview?chr=2L&vc_start=39087872&vc_end=39093776) | Ag.2L.666.0_CDS_at |
| [AGAP006831](http://www.ensembl.org/Anopheles_gambiae/geneview?gene=AGAP006831) | Cuticular protein 57; RR-2 family | [39110972](http://www.ensembl.org/Anopheles_gambiae/contigview?chr=2L&vc_start=39110972&vc_end=39111693) | [39111693](http://www.ensembl.org/Anopheles_gambiae/contigview?chr=2L&vc_start=39110972&vc_end=39111693) | Ag.2L.2127.0_CDS_at |
| [AGAP006832](http://www.ensembl.org/Anopheles_gambiae/geneview?gene=AGAP006832) | structural constituent of eye lens | [39113747](http://www.ensembl.org/Anopheles_gambiae/contigview?chr=2L&vc_start=39113747&vc_end=39114833) | [39114833](http://www.ensembl.org/Anopheles_gambiae/contigview?chr=2L&vc_start=39113747&vc_end=39114833) | Ag.2L.2128.0_CDS_at |
| [AGAP006833](http://www.ensembl.org/Anopheles_gambiae/geneview?gene=AGAP006833) | Cuticular protein 56; RR-2 family | [39116000](http://www.ensembl.org/Anopheles_gambiae/contigview?chr=2L&vc_start=39116000&vc_end=39116582) | [39116582](http://www.ensembl.org/Anopheles_gambiae/contigview?chr=2L&vc_start=39116000&vc_end=39116582) | Ag.2L.2669.0_CDS_at, Ag.2L.2128.0_CDS_at |
| [AGAP006834](http://www.ensembl.org/Anopheles_gambiae/geneview?gene=AGAP006834) | Cuticular protein 69; RR-2 family | [39116981](http://www.ensembl.org/Anopheles_gambiae/contigview?chr=2L&vc_start=39116981&vc_end=39117868) | [39117868](http://www.ensembl.org/Anopheles_gambiae/contigview?chr=2L&vc_start=39116981&vc_end=39117868) | Ag.2L.2669.0_CDS_at, Ag.2L.2128.0_CDS_at |
| [AGAP006835](http://www.ensembl.org/Anopheles_gambiae/geneview?gene=AGAP006835) | DNA binding; nucleus; nuclear envelope | [39124220](http://www.ensembl.org/Anopheles_gambiae/contigview?chr=2L&vc_start=39124220&vc_end=39145770) | [39145770](http://www.ensembl.org/Anopheles_gambiae/contigview?chr=2L&vc_start=39124220&vc_end=39145770) | Ag.2L.592.0_UTR_at, Ag.2L.592.0_CDS_at, Ag.2L.592.0_UTR_at |
| [AGAP006836](http://www.ensembl.org/Anopheles_gambiae/geneview?gene=AGAP006836) | Cuticular protein 101; RR-2 family | [39138827](http://www.ensembl.org/Anopheles_gambiae/contigview?chr=2L&vc_start=39138827&vc_end=39139374) | [39139374](http://www.ensembl.org/Anopheles_gambiae/contigview?chr=2L&vc_start=39138827&vc_end=39139374) |  |
| [AGAP006837](http://www.ensembl.org/Anopheles_gambiae/geneview?gene=AGAP006837) | Cuticular protein 55; RR-2 family | [39151550](http://www.ensembl.org/Anopheles_gambiae/contigview?chr=2L&vc_start=39151550&vc_end=39152078) | [39152078](http://www.ensembl.org/Anopheles_gambiae/contigview?chr=2L&vc_start=39151550&vc_end=39152078) | Ag.2L.2129.0_CDS_at |
| [AGAP006838](http://www.ensembl.org/Anopheles_gambiae/geneview?gene=AGAP006838) | Cuticular protein 68; RR-2 family | [39153888](http://www.ensembl.org/Anopheles_gambiae/contigview?chr=2L&vc_start=39153888&vc_end=39162819) | [39162819](http://www.ensembl.org/Anopheles_gambiae/contigview?chr=2L&vc_start=39153888&vc_end=39162819) | Ag.2L.2130.0_CDS_at |
| [AGAP006839](http://www.ensembl.org/Anopheles_gambiae/geneview?gene=AGAP006839) | Cuticular protein 67; RR-2 family | [39170757](http://www.ensembl.org/Anopheles_gambiae/contigview?chr=2L&vc_start=39170757&vc_end=39171972) | [39171972](http://www.ensembl.org/Anopheles_gambiae/contigview?chr=2L&vc_start=39170757&vc_end=39171972) | Ag.2L.1116.0_CDS_at |
| [AGAP006840](http://www.ensembl.org/Anopheles_gambiae/geneview?gene=AGAP006840) | Cuticular protein 136; RR-2 family | [39174559](http://www.ensembl.org/Anopheles_gambiae/contigview?chr=2L&vc_start=39174559&vc_end=39175063) | [39175063](http://www.ensembl.org/Anopheles_gambiae/contigview?chr=2L&vc_start=39174559&vc_end=39175063) |  |
| [AGAP006841](http://www.ensembl.org/Anopheles_gambiae/geneview?gene=AGAP006841) | Cuticular protein 54; RR-2 family | [39176561](http://www.ensembl.org/Anopheles_gambiae/contigview?chr=2L&vc_start=39176561&vc_end=39177057) | [39177057](http://www.ensembl.org/Anopheles_gambiae/contigview?chr=2L&vc_start=39176561&vc_end=39177057) |  |
| [AGAP006842](http://www.ensembl.org/Anopheles_gambiae/geneview?gene=AGAP006842) | Cuticular protein 53; RR-2 family | [39178730](http://www.ensembl.org/Anopheles_gambiae/contigview?chr=2L&vc_start=39178730&vc_end=39179226) | [39179226](http://www.ensembl.org/Anopheles_gambiae/contigview?chr=2L&vc_start=39178730&vc_end=39179226) | Ag.2L.2670.0_CDS_at |
| [AGAP006843](http://www.ensembl.org/Anopheles_gambiae/geneview?gene=AGAP006843) | Cuticular protein 52; RR-2 family | [39181044](http://www.ensembl.org/Anopheles_gambiae/contigview?chr=2L&vc_start=39181044&vc_end=39181492) | [39181492](http://www.ensembl.org/Anopheles_gambiae/contigview?chr=2L&vc_start=39181044&vc_end=39181492) | Ag.2L.2132.0_CDS_s_at |
| [AGAP006844](http://www.ensembl.org/Anopheles_gambiae/geneview?gene=AGAP006844) | Cuticular protein 51; RR-2 family | [39183142](http://www.ensembl.org/Anopheles_gambiae/contigview?chr=2L&vc_start=39183142&vc_end=39183638) | [39183638](http://www.ensembl.org/Anopheles_gambiae/contigview?chr=2L&vc_start=39183142&vc_end=39183638) | Ag.2L.2132.0_CDS_s_at |
| [AGAP006845](http://www.ensembl.org/Anopheles_gambiae/geneview?gene=AGAP006845) | Cuticular protein 50; RR-2 family | [39185138](http://www.ensembl.org/Anopheles_gambiae/contigview?chr=2L&vc_start=39185138&vc_end=39185622) | [39185622](http://www.ensembl.org/Anopheles_gambiae/contigview?chr=2L&vc_start=39185138&vc_end=39185622) |  |
| [AGAP006846](http://www.ensembl.org/Anopheles_gambiae/geneview?gene=AGAP006846) | Cuticular protein 49; RR-2 family | [39186318](http://www.ensembl.org/Anopheles_gambiae/contigview?chr=2L&vc_start=39186318&vc_end=39186814) | [39186814](http://www.ensembl.org/Anopheles_gambiae/contigview?chr=2L&vc_start=39186318&vc_end=39186814) | Ag.2L.2132.0_CDS_s_at |
| [AGAP006847](http://www.ensembl.org/Anopheles_gambiae/geneview?gene=AGAP006847) | Cuticular protein 48; RR-2 family | [39187823](http://www.ensembl.org/Anopheles_gambiae/contigview?chr=2L&vc_start=39187823&vc_end=39188287) | [39188287](http://www.ensembl.org/Anopheles_gambiae/contigview?chr=2L&vc_start=39187823&vc_end=39188287) | Ag.2L.2132.0_CDS_s_at |
| [AGAP006848](http://www.ensembl.org/Anopheles_gambiae/geneview?gene=AGAP006848) | Cuticular protein 47; RR-2 family | [39189587](http://www.ensembl.org/Anopheles_gambiae/contigview?chr=2L&vc_start=39189587&vc_end=39190043) | [39190043](http://www.ensembl.org/Anopheles_gambiae/contigview?chr=2L&vc_start=39189587&vc_end=39190043) | Ag.2L.2134.0_CDS_at |
| [AGAP006849](http://www.ensembl.org/Anopheles_gambiae/geneview?gene=AGAP006849) | Cuticular protein 46; RR-2 family | [39192163](http://www.ensembl.org/Anopheles_gambiae/contigview?chr=2L&vc_start=39192163&vc_end=39192861) | [39192861](http://www.ensembl.org/Anopheles_gambiae/contigview?chr=2L&vc_start=39192163&vc_end=39192861) |  |
| [AGAP006850](http://www.ensembl.org/Anopheles_gambiae/geneview?gene=AGAP006850) | Cuticular protein 45; RR-2 family | [39194109](http://www.ensembl.org/Anopheles_gambiae/contigview?chr=2L&vc_start=39194109&vc_end=39194498) | [39194498](http://www.ensembl.org/Anopheles_gambiae/contigview?chr=2L&vc_start=39194109&vc_end=39194498) |  |
| [AGAP006851](http://www.ensembl.org/Anopheles_gambiae/geneview?gene=AGAP006851) | Cuticular protein 44; RR-2 family | [39195928](http://www.ensembl.org/Anopheles_gambiae/contigview?chr=2L&vc_start=39195928&vc_end=39196317) | [39196317](http://www.ensembl.org/Anopheles_gambiae/contigview?chr=2L&vc_start=39195928&vc_end=39196317) |  |
| [AGAP006852](http://www.ensembl.org/Anopheles_gambiae/geneview?gene=AGAP006852) | Cuticular protein 43; RR-2 family | [39198204](http://www.ensembl.org/Anopheles_gambiae/contigview?chr=2L&vc_start=39198204&vc_end=39198593) | [39198593](http://www.ensembl.org/Anopheles_gambiae/contigview?chr=2L&vc_start=39198204&vc_end=39198593) |  |
| [AGAP006853](http://www.ensembl.org/Anopheles_gambiae/geneview?gene=AGAP006853) | Cuticular protein 42; RR-2 family | [39200923](http://www.ensembl.org/Anopheles_gambiae/contigview?chr=2L&vc_start=39200923&vc_end=39201294) | [39201294](http://www.ensembl.org/Anopheles_gambiae/contigview?chr=2L&vc_start=39200923&vc_end=39201294) |  |
| [AGAP006854](http://www.ensembl.org/Anopheles_gambiae/geneview?gene=AGAP006854) | Cuticular protein 41; RR-2 family | [39202721](http://www.ensembl.org/Anopheles_gambiae/contigview?chr=2L&vc_start=39202721&vc_end=39203110) | [39203110](http://www.ensembl.org/Anopheles_gambiae/contigview?chr=2L&vc_start=39202721&vc_end=39203110) |  |
| [AGAP006855](http://www.ensembl.org/Anopheles_gambiae/geneview?gene=AGAP006855) | Cuticular protein 40; RR-2 family | [39204665](http://www.ensembl.org/Anopheles_gambiae/contigview?chr=2L&vc_start=39204665&vc_end=39205054) | [39205054](http://www.ensembl.org/Anopheles_gambiae/contigview?chr=2L&vc_start=39204665&vc_end=39205054) |  |
| [AGAP006856](http://www.ensembl.org/Anopheles_gambiae/geneview?gene=AGAP006856) | Cuticular protein 39; RR-2 family | [39207082](http://www.ensembl.org/Anopheles_gambiae/contigview?chr=2L&vc_start=39207082&vc_end=39207468) | [39207468](http://www.ensembl.org/Anopheles_gambiae/contigview?chr=2L&vc_start=39207082&vc_end=39207468) | Ag.2L.2677.0_CDS_at |
| [AGAP006857](http://www.ensembl.org/Anopheles_gambiae/geneview?gene=AGAP006857) | Cuticular protein 38; RR-2 family | [39209289](http://www.ensembl.org/Anopheles_gambiae/contigview?chr=2L&vc_start=39209289&vc_end=39209666) | [39209666](http://www.ensembl.org/Anopheles_gambiae/contigview?chr=2L&vc_start=39209289&vc_end=39209666) |  |
| [AGAP006858](http://www.ensembl.org/Anopheles_gambiae/geneview?gene=AGAP006858) | Cuticular protein 37; RR-2 family | [39211329](http://www.ensembl.org/Anopheles_gambiae/contigview?chr=2L&vc_start=39211329&vc_end=39211784) | [39211784](http://www.ensembl.org/Anopheles_gambiae/contigview?chr=2L&vc_start=39211329&vc_end=39211784) | Ag.2L.2678.0_CDS_at |
| [AGAP006859](http://www.ensembl.org/Anopheles_gambiae/geneview?gene=AGAP006859) | Cuticular protein 66; RR-2 family | [39212866](http://www.ensembl.org/Anopheles_gambiae/contigview?chr=2L&vc_start=39212866&vc_end=39213312) | [39213312](http://www.ensembl.org/Anopheles_gambiae/contigview?chr=2L&vc_start=39212866&vc_end=39213312) |  |
| [AGAP006860](http://www.ensembl.org/Anopheles_gambiae/geneview?gene=AGAP006860) | Cuticular protein 145; RR-2 family | [39214535](http://www.ensembl.org/Anopheles_gambiae/contigview?chr=2L&vc_start=39214535&vc_end=39214978) | [39214978](http://www.ensembl.org/Anopheles_gambiae/contigview?chr=2L&vc_start=39214535&vc_end=39214978) |  |
| [AGAP006861](http://www.ensembl.org/Anopheles_gambiae/geneview?gene=AGAP006861) | Cuticular protein 36; RR-2 family | [39215802](http://www.ensembl.org/Anopheles_gambiae/contigview?chr=2L&vc_start=39215802&vc_end=39216191) | [39216191](http://www.ensembl.org/Anopheles_gambiae/contigview?chr=2L&vc_start=39215802&vc_end=39216191) |  |
| [AGAP006862](http://www.ensembl.org/Anopheles_gambiae/geneview?gene=AGAP006862) | Cuticular protein 35; RR-2 family | [39217701](http://www.ensembl.org/Anopheles_gambiae/contigview?chr=2L&vc_start=39217701&vc_end=39218069) | [39218069](http://www.ensembl.org/Anopheles_gambiae/contigview?chr=2L&vc_start=39217701&vc_end=39218069) |  |
| [AGAP006863](http://www.ensembl.org/Anopheles_gambiae/geneview?gene=AGAP006863) | Cuticular protein 65; RR-2 family | [39219764](http://www.ensembl.org/Anopheles_gambiae/contigview?chr=2L&vc_start=39219764&vc_end=39220196) | [39220196](http://www.ensembl.org/Anopheles_gambiae/contigview?chr=2L&vc_start=39219764&vc_end=39220196) | Ag.2L.2679.0_CDS_at |
| [AGAP006864](http://www.ensembl.org/Anopheles_gambiae/geneview?gene=AGAP006864) | Cuticular protein 34;RR-2 family | [39221590](http://www.ensembl.org/Anopheles_gambiae/contigview?chr=2L&vc_start=39221590&vc_end=39222203) | [39222203](http://www.ensembl.org/Anopheles_gambiae/contigview?chr=2L&vc_start=39221590&vc_end=39222203) | Ag.2L.1714.0_CDS_at, Ag.2L.1456.0_CDS_at |
| [AGAP006865](http://www.ensembl.org/Anopheles_gambiae/geneview?gene=AGAP006865) | Cuticular protein 64; RR-2 family | [39222587](http://www.ensembl.org/Anopheles_gambiae/contigview?chr=2L&vc_start=39222587&vc_end=39223516) | [39223516](http://www.ensembl.org/Anopheles_gambiae/contigview?chr=2L&vc_start=39222587&vc_end=39223516) | Ag.2L.1714.0_CDS_at, Ag.2L.1456.0_CDS_at |
| [AGAP006866](http://www.ensembl.org/Anopheles_gambiae/geneview?gene=AGAP006866) | Cuticular protein 63; RR-2 family | [39226948](http://www.ensembl.org/Anopheles_gambiae/contigview?chr=2L&vc_start=39226948&vc_end=39227651) | [39227651](http://www.ensembl.org/Anopheles_gambiae/contigview?chr=2L&vc_start=39226948&vc_end=39227651) | Ag.2L.1117.0_CDS_at |
| [AGAP006867](http://www.ensembl.org/Anopheles_gambiae/geneview?gene=AGAP006867) | Cuticular protein 141; RR-2 family | [39230211](http://www.ensembl.org/Anopheles_gambiae/contigview?chr=2L&vc_start=39230211&vc_end=39239664) | [39239664](http://www.ensembl.org/Anopheles_gambiae/contigview?chr=2L&vc_start=39230211&vc_end=39239664) | Ag.2L.1457.0_CDS_at |
| [AGAP006868](http://www.ensembl.org/Anopheles_gambiae/geneview?gene=AGAP006868) | Cuticular protein 140; RR-2 family | [39269028](http://www.ensembl.org/Anopheles_gambiae/contigview?chr=2L&vc_start=39269028&vc_end=39275406) | [39275406](http://www.ensembl.org/Anopheles_gambiae/contigview?chr=2L&vc_start=39269028&vc_end=39275406) | Ag.2L.2142.1_a_at, Ag.2L.3526.0_at, U50476.1_s_at |
| [AGAP006869](http://www.ensembl.org/Anopheles_gambiae/geneview?gene=AGAP006869) | trypsin | [39278059](http://www.ensembl.org/Anopheles_gambiae/contigview?chr=2L&vc_start=39278059&vc_end=39279025) | [39279025](http://www.ensembl.org/Anopheles_gambiae/contigview?chr=2L&vc_start=39278059&vc_end=39279025) | Ag.2L.2680.0_CDS_at |
| [AGAP006870](http://www.ensembl.org/Anopheles_gambiae/geneview?gene=AGAP006870) | protein binding | [39282567](http://www.ensembl.org/Anopheles_gambiae/contigview?chr=2L&vc_start=39282567&vc_end=39283388) | [39283388](http://www.ensembl.org/Anopheles_gambiae/contigview?chr=2L&vc_start=39282567&vc_end=39283388) | Ag.2L.2144.0_CDS_at |
| [AGAP006871](http://www.ensembl.org/Anopheles_gambiae/geneview?gene=AGAP006871) | ribosomal protein S4E | [39285040](http://www.ensembl.org/Anopheles_gambiae/contigview?chr=2L&vc_start=39285040&vc_end=39287251) | [39287251](http://www.ensembl.org/Anopheles_gambiae/contigview?chr=2L&vc_start=39285040&vc_end=39287251) | Ag.2L.149.0_CDS_at |
| [AGAP006872](http://www.ensembl.org/Anopheles_gambiae/geneview?gene=AGAP006872) | nim-A-like kinase; receptor signaling | [39287864](http://www.ensembl.org/Anopheles_gambiae/contigview?chr=2L&vc_start=39287864&vc_end=39300071) | [39300071](http://www.ensembl.org/Anopheles_gambiae/contigview?chr=2L&vc_start=39287864&vc_end=39300071) | Ag.2L.2145.0_CDS_at |
| [AGAP006873](http://www.ensembl.org/Anopheles_gambiae/geneview?gene=AGAP006873) | GTPase | [39302116](http://www.ensembl.org/Anopheles_gambiae/contigview?chr=2L&vc_start=39302116&vc_end=39306607) | [39306607](http://www.ensembl.org/Anopheles_gambiae/contigview?chr=2L&vc_start=39302116&vc_end=39306607) | Ag.2L.2681.0_CDS_at |
| [AGAP006874](http://www.ensembl.org/Anopheles_gambiae/geneview?gene=AGAP006874) | gustatory receptor GPRGR29 | [39308977](http://www.ensembl.org/Anopheles_gambiae/contigview?chr=2L&vc_start=39308977&vc_end=39310512) | [39310512](http://www.ensembl.org/Anopheles_gambiae/contigview?chr=2L&vc_start=39308977&vc_end=39310512) | Ag.2L.106.0_CDS_at, Ag.2L.107.0_CDS_at |
| [AGAP006875](http://www.ensembl.org/Anopheles_gambiae/geneview?gene=AGAP006875) | gustatory receptor GPRGR30 | [39310745](http://www.ensembl.org/Anopheles_gambiae/contigview?chr=2L&vc_start=39310745&vc_end=39312218) | [39312218](http://www.ensembl.org/Anopheles_gambiae/contigview?chr=2L&vc_start=39310745&vc_end=39312218) | Ag.2L.108.0_CDS_at, Ag.2L.107.0_CDS_at |
| [AGAP006876](http://www.ensembl.org/Anopheles_gambiae/geneview?gene=AGAP006876) | gustatory receptor GPRGR31 | [39312570](http://www.ensembl.org/Anopheles_gambiae/contigview?chr=2L&vc_start=39312570&vc_end=39314187) | [39314187](http://www.ensembl.org/Anopheles_gambiae/contigview?chr=2L&vc_start=39312570&vc_end=39314187) | Ag.2L.108.0_CDS_at |
| [AGAP006877](http://www.ensembl.org/Anopheles_gambiae/geneview?gene=AGAP006877) | gustatory receptor GPRGR32 | [39314564](http://www.ensembl.org/Anopheles_gambiae/contigview?chr=2L&vc_start=39314564&vc_end=39318160) | [39318160](http://www.ensembl.org/Anopheles_gambiae/contigview?chr=2L&vc_start=39314564&vc_end=39318160) | Ag.2L.109.0_CDS_a_at, Ag.2L.499.1_s_at |
| [AGAP006878](http://www.ensembl.org/Anopheles_gambiae/geneview?gene=AGAP006878) |  | [39318226](http://www.ensembl.org/Anopheles_gambiae/contigview?chr=2L&vc_start=39318226&vc_end=39319485) | [39319485](http://www.ensembl.org/Anopheles_gambiae/contigview?chr=2L&vc_start=39318226&vc_end=39319485) | Ag.2L.109.0_CDS_a_at, Ag.2L.499.1_s_at |
| [AGAP006879](http://www.ensembl.org/Anopheles_gambiae/geneview?gene=AGAP006879) | proton-transporting ATP synthase | [39319780](http://www.ensembl.org/Anopheles_gambiae/contigview?chr=2L&vc_start=39319780&vc_end=39320739) | [39320739](http://www.ensembl.org/Anopheles_gambiae/contigview?chr=2L&vc_start=39319780&vc_end=39320739) | Ag.2L.222.1_a_at |
| [AGAP006880](http://www.ensembl.org/Anopheles_gambiae/geneview?gene=AGAP006880) | lipocalin-1 interacting membrane receptor | [39321706](http://www.ensembl.org/Anopheles_gambiae/contigview?chr=2L&vc_start=39321706&vc_end=39337266) | [39337266](http://www.ensembl.org/Anopheles_gambiae/contigview?chr=2L&vc_start=39321706&vc_end=39337266) | Ag.2L.1118.1_CDS_a_at |
| [AGAP006881](http://www.ensembl.org/Anopheles_gambiae/geneview?gene=AGAP006881) | polypeptide N-acetylgalactosaminyl transferase | [39325101](http://www.ensembl.org/Anopheles_gambiae/contigview?chr=2L&vc_start=39325101&vc_end=39327080) | [39327080](http://www.ensembl.org/Anopheles_gambiae/contigview?chr=2L&vc_start=39325101&vc_end=39327080) | Ag.2L.2682.0_CDS_at |
| [AGAP006882](http://www.ensembl.org/Anopheles_gambiae/geneview?gene=AGAP006882) |  | [39334625](http://www.ensembl.org/Anopheles_gambiae/contigview?chr=2L&vc_start=39334625&vc_end=39335592) | [39335592](http://www.ensembl.org/Anopheles_gambiae/contigview?chr=2L&vc_start=39334625&vc_end=39335592) |  |
| [AGAP006883](http://www.ensembl.org/Anopheles_gambiae/geneview?gene=AGAP006883) | protein binding | [39342320](http://www.ensembl.org/Anopheles_gambiae/contigview?chr=2L&vc_start=39342320&vc_end=39345905) | [39345905](http://www.ensembl.org/Anopheles_gambiae/contigview?chr=2L&vc_start=39342320&vc_end=39345905) | Ag.2L.2683.1_at |
| [AGAP006884](http://www.ensembl.org/Anopheles_gambiae/geneview?gene=AGAP006884) | golgin subfamily A; vesicle-mediated transport | [39347620](http://www.ensembl.org/Anopheles_gambiae/contigview?chr=2L&vc_start=39347620&vc_end=39354291) | [39354291](http://www.ensembl.org/Anopheles_gambiae/contigview?chr=2L&vc_start=39347620&vc_end=39354291) | Ag.2L.2683.1_at, Ag.2L.2683.0_CDS_at |
| [AGAP006885](http://www.ensembl.org/Anopheles_gambiae/geneview?gene=AGAP006885) | nuclear mRNA splicing | [39355321](http://www.ensembl.org/Anopheles_gambiae/contigview?chr=2L&vc_start=39355321&vc_end=39363783) | [39363783](http://www.ensembl.org/Anopheles_gambiae/contigview?chr=2L&vc_start=39355321&vc_end=39363783) | Ag.2L.2146.0_CDS_at, Ag.2L.526.0_CDS_at |
| [AGAP006886](http://www.ensembl.org/Anopheles_gambiae/geneview?gene=AGAP006886) | tRNA-Glu | [39363918](http://www.ensembl.org/Anopheles_gambiae/contigview?chr=2L&vc_start=39363918&vc_end=39363989) | [39363989](http://www.ensembl.org/Anopheles_gambiae/contigview?chr=2L&vc_start=39363918&vc_end=39363989) | Ag.2L.2146.0_CDS_at |
| [AGAP006887](http://www.ensembl.org/Anopheles_gambiae/geneview?gene=AGAP006887) | microtubule-based movement; ATPase | [39364952](http://www.ensembl.org/Anopheles_gambiae/contigview?chr=2L&vc_start=39364952&vc_end=39365620) | [39365620](http://www.ensembl.org/Anopheles_gambiae/contigview?chr=2L&vc_start=39364952&vc_end=39365620) | Ag.2L.2146.0_CDS_at |
| [AGAP006888](http://www.ensembl.org/Anopheles_gambiae/geneview?gene=AGAP006888) | ARF GTPase activator | [39367809](http://www.ensembl.org/Anopheles_gambiae/contigview?chr=2L&vc_start=39367809&vc_end=39371332) | [39371332](http://www.ensembl.org/Anopheles_gambiae/contigview?chr=2L&vc_start=39367809&vc_end=39371332) | Ag.2L.1715.0_CDS_at |
| [AGAP006889](http://www.ensembl.org/Anopheles_gambiae/geneview?gene=AGAP006889) | RNA pol II transcription elongation factor | [39373037](http://www.ensembl.org/Anopheles_gambiae/contigview?chr=2L&vc_start=39373037&vc_end=39375732) | [39375732](http://www.ensembl.org/Anopheles_gambiae/contigview?chr=2L&vc_start=39373037&vc_end=39375732) | Ag.2L.919.0_CDS_at, Ag.2L.1458.0_CDS_at |
| [AGAP006890](http://www.ensembl.org/Anopheles_gambiae/geneview?gene=AGAP006890) | ADP-ribosylation factor 6 | [39376198](http://www.ensembl.org/Anopheles_gambiae/contigview?chr=2L&vc_start=39376198&vc_end=39410515) | [39410515](http://www.ensembl.org/Anopheles_gambiae/contigview?chr=2L&vc_start=39376198&vc_end=39410515) | Ag.2L.919.0_CDS_at, Ag.2L.920.0_CDS_at |
| [AGAP006891](http://www.ensembl.org/Anopheles_gambiae/geneview?gene=AGAP006891) | NADH dehydrogenase | [39426797](http://www.ensembl.org/Anopheles_gambiae/contigview?chr=2L&vc_start=39426797&vc_end=39428212) | [39428212](http://www.ensembl.org/Anopheles_gambiae/contigview?chr=2L&vc_start=39426797&vc_end=39428212) | Ag.2L.2684.0_CDS_at |
| [AGAP006892](http://www.ensembl.org/Anopheles_gambiae/geneview?gene=AGAP006892) | DNA binding; hemostasis | [39472033](http://www.ensembl.org/Anopheles_gambiae/contigview?chr=2L&vc_start=39472033&vc_end=39475207) | [39475207](http://www.ensembl.org/Anopheles_gambiae/contigview?chr=2L&vc_start=39472033&vc_end=39475207) | Ag.2L.1459.0_CDS_at |
| [AGAP006893](http://www.ensembl.org/Anopheles_gambiae/geneview?gene=AGAP006893) | actin binding; wing morphogenesis | [39550574](http://www.ensembl.org/Anopheles_gambiae/contigview?chr=2L&vc_start=39550574&vc_end=39551848) | [39551848](http://www.ensembl.org/Anopheles_gambiae/contigview?chr=2L&vc_start=39550574&vc_end=39551848) | Ag.2L.2148.0_CDS_at |
| [AGAP006894](http://www.ensembl.org/Anopheles_gambiae/geneview?gene=AGAP006894) | dimethylallyltransferase | [39556088](http://www.ensembl.org/Anopheles_gambiae/contigview?chr=2L&vc_start=39556088&vc_end=39558181) | [39558181](http://www.ensembl.org/Anopheles_gambiae/contigview?chr=2L&vc_start=39556088&vc_end=39558181) | Ag.2L.1261.0_CDS_at |
| [AGAP006895](http://www.ensembl.org/Anopheles_gambiae/geneview?gene=AGAP006895) | signal recognition particle protein 19; 7SRNA-binding | [39558794](http://www.ensembl.org/Anopheles_gambiae/contigview?chr=2L&vc_start=39558794&vc_end=39559529) | [39559529](http://www.ensembl.org/Anopheles_gambiae/contigview?chr=2L&vc_start=39558794&vc_end=39559529) | Ag.2L.921.0_CDS_s_at, Ag.2L.2686.0_CDS_at |
| [AGAP006896](http://www.ensembl.org/Anopheles_gambiae/geneview?gene=AGAP006896) |  | [39560771](http://www.ensembl.org/Anopheles_gambiae/contigview?chr=2L&vc_start=39560771&vc_end=39561502) | [39561502](http://www.ensembl.org/Anopheles_gambiae/contigview?chr=2L&vc_start=39560771&vc_end=39561502) | Ag.2L.921.0_CDS_s_at, Ag.2L.2686.0_CDS_at |
| [AGAP006897](http://www.ensembl.org/Anopheles_gambiae/geneview?gene=AGAP006897) | GABA-A receptor epsilon-like subunit | [39569238](http://www.ensembl.org/Anopheles_gambiae/contigview?chr=2L&vc_start=39569238&vc_end=39571572) | [39571572](http://www.ensembl.org/Anopheles_gambiae/contigview?chr=2L&vc_start=39569238&vc_end=39571572) | Ag.2L.2149.0_CDS_at |
| [AGAP006898](http://www.ensembl.org/Anopheles_gambiae/geneview?gene=AGAP006898) | chitinase | [39574076](http://www.ensembl.org/Anopheles_gambiae/contigview?chr=2L&vc_start=39574076&vc_end=39578295) | [39578295](http://www.ensembl.org/Anopheles_gambiae/contigview?chr=2L&vc_start=39574076&vc_end=39578295) | Ag.2L.1716.0_CDS_at |
| [AGAP006899](http://www.ensembl.org/Anopheles_gambiae/geneview?gene=AGAP006899) | salivary secreted peptide | [39585632](http://www.ensembl.org/Anopheles_gambiae/contigview?chr=2L&vc_start=39585632&vc_end=39586107) | [39586107](http://www.ensembl.org/Anopheles_gambiae/contigview?chr=2L&vc_start=39585632&vc_end=39586107) |  |
| [AGAP006900](http://www.ensembl.org/Anopheles_gambiae/geneview?gene=AGAP006900) | possible ATP-dependent protease | [39634589](http://www.ensembl.org/Anopheles_gambiae/contigview?chr=2L&vc_start=39634589&vc_end=39672322) | [39672322](http://www.ensembl.org/Anopheles_gambiae/contigview?chr=2L&vc_start=39634589&vc_end=39672322) | Ag.2L.2688.0_CDS_at |
| [AGAP006901](http://www.ensembl.org/Anopheles_gambiae/geneview?gene=AGAP006901) | protein binding | [39704624](http://www.ensembl.org/Anopheles_gambiae/contigview?chr=2L&vc_start=39704624&vc_end=39749608) | [39749608](http://www.ensembl.org/Anopheles_gambiae/contigview?chr=2L&vc_start=39704624&vc_end=39749608) | Ag.2L.2689.2_a_at, Ag.2L.355.0_CDS_at, Ag.2L.355.0_UTR_at, Ag.2L.1717.0_CDS_at |
| [AGAP006902](http://www.ensembl.org/Anopheles_gambiae/geneview?gene=AGAP006902) |  | [39754475](http://www.ensembl.org/Anopheles_gambiae/contigview?chr=2L&vc_start=39754475&vc_end=39757719) | [39757719](http://www.ensembl.org/Anopheles_gambiae/contigview?chr=2L&vc_start=39754475&vc_end=39757719) | Ag.2L.2150.0_CDS_at |
| [AGAP006903](http://www.ensembl.org/Anopheles_gambiae/geneview?gene=AGAP006903) | Alpha-2,6-sialyltransferase; glycosylation | [39809855](http://www.ensembl.org/Anopheles_gambiae/contigview?chr=2L&vc_start=39809855&vc_end=39812317) | [39812317](http://www.ensembl.org/Anopheles_gambiae/contigview?chr=2L&vc_start=39809855&vc_end=39812317) | Ag.2L.1119.0_CDS_at |
| [AGAP006904](http://www.ensembl.org/Anopheles_gambiae/geneview?gene=AGAP006904) | metalloendopeptidase | [39815884](http://www.ensembl.org/Anopheles_gambiae/contigview?chr=2L&vc_start=39815884&vc_end=39838118) | [39838118](http://www.ensembl.org/Anopheles_gambiae/contigview?chr=2L&vc_start=39815884&vc_end=39838118) | Ag.2L.414.0_CDS_a_at, Ag.2L.414.0_UTR_a_at |
| [AGAP006905](http://www.ensembl.org/Anopheles_gambiae/geneview?gene=AGAP006905) |  | [39840850](http://www.ensembl.org/Anopheles_gambiae/contigview?chr=2L&vc_start=39840850&vc_end=39847803) | [39847803](http://www.ensembl.org/Anopheles_gambiae/contigview?chr=2L&vc_start=39840850&vc_end=39847803) | Ag.2L.1120.0_CDS_at |
| [AGAP006906](http://www.ensembl.org/Anopheles_gambiae/geneview?gene=AGAP006906) | adenosine deaminase | [39851960](http://www.ensembl.org/Anopheles_gambiae/contigview?chr=2L&vc_start=39851960&vc_end=39854636) | [39854636](http://www.ensembl.org/Anopheles_gambiae/contigview?chr=2L&vc_start=39851960&vc_end=39854636) | Ag.2L.1121.0_CDS_at |
| [AGAP006907](http://www.ensembl.org/Anopheles_gambiae/geneview?gene=AGAP006907) | adenosine deaminase | [39856602](http://www.ensembl.org/Anopheles_gambiae/contigview?chr=2L&vc_start=39856602&vc_end=39858724) | [39858724](http://www.ensembl.org/Anopheles_gambiae/contigview?chr=2L&vc_start=39856602&vc_end=39858724) | Ag.2L.778.0_CDS_at |
| [AGAP006908](http://www.ensembl.org/Anopheles_gambiae/geneview?gene=AGAP006908) | growth factor | [39873667](http://www.ensembl.org/Anopheles_gambiae/contigview?chr=2L&vc_start=39873667&vc_end=39878129) | [39878129](http://www.ensembl.org/Anopheles_gambiae/contigview?chr=2L&vc_start=39873667&vc_end=39878129) | Ag.2L.2690.0_CDS_at |
| [AGAP006909](http://www.ensembl.org/Anopheles_gambiae/geneview?gene=AGAP006909) | Serine Protease Inhibitor SRPN1 | [39889421](http://www.ensembl.org/Anopheles_gambiae/contigview?chr=2L&vc_start=39889421&vc_end=39894237) | [39894237](http://www.ensembl.org/Anopheles_gambiae/contigview?chr=2L&vc_start=39889421&vc_end=39894237) | Ag.2L.54.0_CDS_a_at, Ag.2L.110.1_a_at |
| [AGAP006910](http://www.ensembl.org/Anopheles_gambiae/geneview?gene=AGAP006910) | Serine Protease Inhibitor SRPN3 | [39894816](http://www.ensembl.org/Anopheles_gambiae/contigview?chr=2L&vc_start=39894816&vc_end=39896384) | [39896384](http://www.ensembl.org/Anopheles_gambiae/contigview?chr=2L&vc_start=39894816&vc_end=39896384) | Ag.2L.110.1_a_at |
| [AGAP006911](http://www.ensembl.org/Anopheles_gambiae/geneview?gene=AGAP006911) | Serine Protease Inhibitor SRPN2 | [39896875](http://www.ensembl.org/Anopheles_gambiae/contigview?chr=2L&vc_start=39896875&vc_end=39899752) | [39899752](http://www.ensembl.org/Anopheles_gambiae/contigview?chr=2L&vc_start=39896875&vc_end=39899752) | AF203339.1_at, Ag.2L.14.0_CDS_at, AF203339.1_s_at |
| [AGAP006912](http://www.ensembl.org/Anopheles_gambiae/geneview?gene=AGAP006912) |  | [39923807](http://www.ensembl.org/Anopheles_gambiae/contigview?chr=2L&vc_start=39923807&vc_end=39926229) | [39926229](http://www.ensembl.org/Anopheles_gambiae/contigview?chr=2L&vc_start=39923807&vc_end=39926229) |  |
| [AGAP006913](http://www.ensembl.org/Anopheles_gambiae/geneview?gene=AGAP006913) | receptor | [39948848](http://www.ensembl.org/Anopheles_gambiae/contigview?chr=2L&vc_start=39948848&vc_end=39951539) | [39951539](http://www.ensembl.org/Anopheles_gambiae/contigview?chr=2L&vc_start=39948848&vc_end=39951539) | Ag.2L.2691.0_CDS_at |
| [AGAP006914](http://www.ensembl.org/Anopheles_gambiae/geneview?gene=AGAP006914) | angiopoietin-related | [39966752](http://www.ensembl.org/Anopheles_gambiae/contigview?chr=2L&vc_start=39966752&vc_end=39968185) | [39968185](http://www.ensembl.org/Anopheles_gambiae/contigview?chr=2L&vc_start=39966752&vc_end=39968185) | Ag.2L.1460.0_CDS_at |
| [AGAP006915](http://www.ensembl.org/Anopheles_gambiae/geneview?gene=AGAP006915) | low density lipoprotein receptor | [39991721](http://www.ensembl.org/Anopheles_gambiae/contigview?chr=2L&vc_start=39991721&vc_end=39993168) | [39993168](http://www.ensembl.org/Anopheles_gambiae/contigview?chr=2L&vc_start=39991721&vc_end=39993168) | Ag.2L.779.0_CDS_at, Ag.2L.2151.0_CDS_at |
| [AGAP006916](http://www.ensembl.org/Anopheles_gambiae/geneview?gene=AGAP006916) |  | [39993541](http://www.ensembl.org/Anopheles_gambiae/contigview?chr=2L&vc_start=39993541&vc_end=39994220) | [39994220](http://www.ensembl.org/Anopheles_gambiae/contigview?chr=2L&vc_start=39993541&vc_end=39994220) | Ag.2L.2151.0_CDS_at |
| [AGAP006917](http://www.ensembl.org/Anopheles_gambiae/geneview?gene=AGAP006917) | gustatory receptor GPRGR55 | [39994248](http://www.ensembl.org/Anopheles_gambiae/contigview?chr=2L&vc_start=39994248&vc_end=39995592) | [39995592](http://www.ensembl.org/Anopheles_gambiae/contigview?chr=2L&vc_start=39994248&vc_end=39995592) | Ag.2L.779.0_CDS_at, Ag.2L.2151.0_CDS_at |
| [AGAP006918](http://www.ensembl.org/Anopheles_gambiae/geneview?gene=AGAP006918) | NADH dehydrogenase | [39995907](http://www.ensembl.org/Anopheles_gambiae/contigview?chr=2L&vc_start=39995907&vc_end=39997107) | [39997107](http://www.ensembl.org/Anopheles_gambiae/contigview?chr=2L&vc_start=39995907&vc_end=39997107) | Ag.2L.217.0_CDS_a_at |
| [AGAP006919](http://www.ensembl.org/Anopheles_gambiae/geneview?gene=AGAP006919) |  | [40002072](http://www.ensembl.org/Anopheles_gambiae/contigview?chr=2L&vc_start=40002072&vc_end=40004048) | [40004048](http://www.ensembl.org/Anopheles_gambiae/contigview?chr=2L&vc_start=40002072&vc_end=40004048) | Ag.2L.1461.1_a_at |
| [AGAP006920](http://www.ensembl.org/Anopheles_gambiae/geneview?gene=AGAP006920) |  | [40004448](http://www.ensembl.org/Anopheles_gambiae/contigview?chr=2L&vc_start=40004448&vc_end=40005004) | [40005004](http://www.ensembl.org/Anopheles_gambiae/contigview?chr=2L&vc_start=40004448&vc_end=40005004) |  |
| [AGAP006921](http://www.ensembl.org/Anopheles_gambiae/geneview?gene=AGAP006921) | CED-6/GULP; signal transduction | [40025650](http://www.ensembl.org/Anopheles_gambiae/contigview?chr=2L&vc_start=40025650&vc_end=40030767) | [40030767](http://www.ensembl.org/Anopheles_gambiae/contigview?chr=2L&vc_start=40025650&vc_end=40030767) | Ag.2L.3095.0_at, Ag.2L.780.0_CDS_at, Ag.2L.780.1_at |
| [AGAP006922](http://www.ensembl.org/Anopheles_gambiae/geneview?gene=AGAP006922) | tRNA-Ser | [40037637](http://www.ensembl.org/Anopheles_gambiae/contigview?chr=2L&vc_start=40037637&vc_end=40037718) | [40037718](http://www.ensembl.org/Anopheles_gambiae/contigview?chr=2L&vc_start=40037637&vc_end=40037718) |  |
| [AGAP006923](http://www.ensembl.org/Anopheles_gambiae/geneview?gene=AGAP006923) | RNA polymerase II transcription factor | [40044234](http://www.ensembl.org/Anopheles_gambiae/contigview?chr=2L&vc_start=40044234&vc_end=40051309) | [40051309](http://www.ensembl.org/Anopheles_gambiae/contigview?chr=2L&vc_start=40044234&vc_end=40051309) | Ag.2L.2692.0_CDS_at |
| [AGAP006924](http://www.ensembl.org/Anopheles_gambiae/geneview?gene=AGAP006924) | protein binding | [40059121](http://www.ensembl.org/Anopheles_gambiae/contigview?chr=2L&vc_start=40059121&vc_end=40059696) | [40059696](http://www.ensembl.org/Anopheles_gambiae/contigview?chr=2L&vc_start=40059121&vc_end=40059696) | Ag.2L.1718.0_CDS_a_at |
| [AGAP006925](http://www.ensembl.org/Anopheles_gambiae/geneview?gene=AGAP006925) | polypeptide N-acetylgalactosaminyl transferase | [40062068](http://www.ensembl.org/Anopheles_gambiae/contigview?chr=2L&vc_start=40062068&vc_end=40076345) | [40076345](http://www.ensembl.org/Anopheles_gambiae/contigview?chr=2L&vc_start=40062068&vc_end=40076345) | Ag.2L.1720.0_CDS_at, Ag.2L.1719.0_CDS_at |
| [AGAP006926](http://www.ensembl.org/Anopheles_gambiae/geneview?gene=AGAP006926) | oxidoreductase | [40078617](http://www.ensembl.org/Anopheles_gambiae/contigview?chr=2L&vc_start=40078617&vc_end=40079751) | [40079751](http://www.ensembl.org/Anopheles_gambiae/contigview?chr=2L&vc_start=40078617&vc_end=40079751) | Ag.2L.1721.0_CDS_at |
| [AGAP006927](http://www.ensembl.org/Anopheles_gambiae/geneview?gene=AGAP006927) | RNA polymerase II transcription factor | [40081078](http://www.ensembl.org/Anopheles_gambiae/contigview?chr=2L&vc_start=40081078&vc_end=40082362) | [40082362](http://www.ensembl.org/Anopheles_gambiae/contigview?chr=2L&vc_start=40081078&vc_end=40082362) | Ag.2L.561.0_CDS_at |
| [AGAP006928](http://www.ensembl.org/Anopheles_gambiae/geneview?gene=AGAP006928) |  | [40087894](http://www.ensembl.org/Anopheles_gambiae/contigview?chr=2L&vc_start=40087894&vc_end=40088638) | [40088638](http://www.ensembl.org/Anopheles_gambiae/contigview?chr=2L&vc_start=40087894&vc_end=40088638) | Ag.2L.1722.0_CDS_at |
| [AGAP006929](http://www.ensembl.org/Anopheles_gambiae/geneview?gene=AGAP006929) |  | [40095187](http://www.ensembl.org/Anopheles_gambiae/contigview?chr=2L&vc_start=40095187&vc_end=40095615) | [40095615](http://www.ensembl.org/Anopheles_gambiae/contigview?chr=2L&vc_start=40095187&vc_end=40095615) |  |
| [AGAP006930](http://www.ensembl.org/Anopheles_gambiae/geneview?gene=AGAP006930) | zinc finger transcription factor | [40104182](http://www.ensembl.org/Anopheles_gambiae/contigview?chr=2L&vc_start=40104182&vc_end=40105484) | [40105484](http://www.ensembl.org/Anopheles_gambiae/contigview?chr=2L&vc_start=40104182&vc_end=40105484) | Ag.2L.2152.0_CDS_at |
| [AGAP006931](http://www.ensembl.org/Anopheles_gambiae/geneview?gene=AGAP006931) | Cuticular protein 111; RR-3 family | [40110606](http://www.ensembl.org/Anopheles_gambiae/contigview?chr=2L&vc_start=40110606&vc_end=40111688) | [40111688](http://www.ensembl.org/Anopheles_gambiae/contigview?chr=2L&vc_start=40110606&vc_end=40111688) | Ag.2L.2153.0_CDS_at |
| [AGAP006932](http://www.ensembl.org/Anopheles_gambiae/geneview?gene=AGAP006932) | serine/threonine kinase; cytokinesis | [40113355](http://www.ensembl.org/Anopheles_gambiae/contigview?chr=2L&vc_start=40113355&vc_end=40120723) | [40120723](http://www.ensembl.org/Anopheles_gambiae/contigview?chr=2L&vc_start=40113355&vc_end=40120723) | Ag.2L.1262.0_CDS_at |
| [AGAP006933](http://www.ensembl.org/Anopheles_gambiae/geneview?gene=AGAP006933) |  | [40121383](http://www.ensembl.org/Anopheles_gambiae/contigview?chr=2L&vc_start=40121383&vc_end=40123629) | [40123629](http://www.ensembl.org/Anopheles_gambiae/contigview?chr=2L&vc_start=40121383&vc_end=40123629) | Ag.2L.922.0_CDS_at |
| [AGAP006934](http://www.ensembl.org/Anopheles_gambiae/geneview?gene=AGAP006934) |  | [40127227](http://www.ensembl.org/Anopheles_gambiae/contigview?chr=2L&vc_start=40127227&vc_end=40128852) | [40128852](http://www.ensembl.org/Anopheles_gambiae/contigview?chr=2L&vc_start=40127227&vc_end=40128852) | Ag.2L.2693.0_CDS_at |
| [AGAP006935](http://www.ensembl.org/Anopheles_gambiae/geneview?gene=AGAP006935) | transcription factor complex | [40147693](http://www.ensembl.org/Anopheles_gambiae/contigview?chr=2L&vc_start=40147693&vc_end=40156760) | [40156760](http://www.ensembl.org/Anopheles_gambiae/contigview?chr=2L&vc_start=40147693&vc_end=40156760) | Ag.2L.2863.0_at, Ag.2L.1263.1_a_at |
| [AGAP006936](http://www.ensembl.org/Anopheles_gambiae/geneview?gene=AGAP006936) | oxidative phosphorylation; electron transport | [40157852](http://www.ensembl.org/Anopheles_gambiae/contigview?chr=2L&vc_start=40157852&vc_end=40161112) | [40161112](http://www.ensembl.org/Anopheles_gambiae/contigview?chr=2L&vc_start=40157852&vc_end=40161112) | Ag.2L.160.4_CDS_a_at, Ag.2L.160.0_UTR_a_at, Ag.2L.160.0_CDS_a_at |
| [AGAP006937](http://www.ensembl.org/Anopheles_gambiae/geneview?gene=AGAP006937) | ubiquitin conjugating enzyme | [40163100](http://www.ensembl.org/Anopheles_gambiae/contigview?chr=2L&vc_start=40163100&vc_end=40166886) | [40166886](http://www.ensembl.org/Anopheles_gambiae/contigview?chr=2L&vc_start=40163100&vc_end=40166886) | Ag.2L.562.0_CDS_a_at, Ag.2L.562.0_UTR_a_at |
| [AGAP006938](http://www.ensembl.org/Anopheles_gambiae/geneview?gene=AGAP006938) | S-adenosylmethionine-dependent methyltransferase | [40167174](http://www.ensembl.org/Anopheles_gambiae/contigview?chr=2L&vc_start=40167174&vc_end=40169246) | [40169246](http://www.ensembl.org/Anopheles_gambiae/contigview?chr=2L&vc_start=40167174&vc_end=40169246) | Ag.2L.1264.0_CDS_at |
| [AGAP006939](http://www.ensembl.org/Anopheles_gambiae/geneview?gene=AGAP006939) | RNA/DNA helicase; RNAi | [40169708](http://www.ensembl.org/Anopheles_gambiae/contigview?chr=2L&vc_start=40169708&vc_end=40175624) | [40175624](http://www.ensembl.org/Anopheles_gambiae/contigview?chr=2L&vc_start=40169708&vc_end=40175624) | Ag.2L.923.0_UTR_at, Ag.2L.3181.0_at, Ag.2L.2818.1_at, Ag.2L.923.0_CDS_at |
| [AGAP006940](http://www.ensembl.org/Anopheles_gambiae/geneview?gene=AGAP006940) |  | [40177771](http://www.ensembl.org/Anopheles_gambiae/contigview?chr=2L&vc_start=40177771&vc_end=40178877) | [40178877](http://www.ensembl.org/Anopheles_gambiae/contigview?chr=2L&vc_start=40177771&vc_end=40178877) | Ag.2L.923.0_CDS_at, Ag.2L.2694.0_CDS_at |
| [AGAP006941](http://www.ensembl.org/Anopheles_gambiae/geneview?gene=AGAP006941) | dead box; ATP-dependent RNA helicase | [40180268](http://www.ensembl.org/Anopheles_gambiae/contigview?chr=2L&vc_start=40180268&vc_end=40182471) | [40182471](http://www.ensembl.org/Anopheles_gambiae/contigview?chr=2L&vc_start=40180268&vc_end=40182471) | Ag.2L.1265.1_CDS_a_at |
| [AGAP006942](http://www.ensembl.org/Anopheles_gambiae/geneview?gene=AGAP006942) | importin-4; protein-nucleus import | [40183406](http://www.ensembl.org/Anopheles_gambiae/contigview?chr=2L&vc_start=40183406&vc_end=40190226) | [40190226](http://www.ensembl.org/Anopheles_gambiae/contigview?chr=2L&vc_start=40183406&vc_end=40190226) | Ag.2L.781.0_CDS_at, Ag.2L.1723.0_CDS_at |
| [AGAP006943](http://www.ensembl.org/Anopheles_gambiae/geneview?gene=AGAP006943) | acetyl CoA transporter | [40190504](http://www.ensembl.org/Anopheles_gambiae/contigview?chr=2L&vc_start=40190504&vc_end=40192853) | [40192853](http://www.ensembl.org/Anopheles_gambiae/contigview?chr=2L&vc_start=40190504&vc_end=40192853) | Ag.2L.1723.0_CDS_at |
| [AGAP006944](http://www.ensembl.org/Anopheles_gambiae/geneview?gene=AGAP006944) | translation initiation factor | [40193242](http://www.ensembl.org/Anopheles_gambiae/contigview?chr=2L&vc_start=40193242&vc_end=40195285) | [40195285](http://www.ensembl.org/Anopheles_gambiae/contigview?chr=2L&vc_start=40193242&vc_end=40195285) | Ag.2L.214.0_CDS_at |
| [AGAP006945](http://www.ensembl.org/Anopheles_gambiae/geneview?gene=AGAP006945) | DNA helicase; repair, recombination | [40195615](http://www.ensembl.org/Anopheles_gambiae/contigview?chr=2L&vc_start=40195615&vc_end=40198230) | [40198230](http://www.ensembl.org/Anopheles_gambiae/contigview?chr=2L&vc_start=40195615&vc_end=40198230) | Ag.2L.1462.1_a_at, Ag.2L.2154.0_CDS_at, Ag.2L.1012.0_CDS_at |
| [AGAP006946](http://www.ensembl.org/Anopheles_gambiae/geneview?gene=AGAP006946) | chaperone binding; “de novo” protein folding; prefoldin complex | [40198251](http://www.ensembl.org/Anopheles_gambiae/contigview?chr=2L&vc_start=40198251&vc_end=40198881) | [40198881](http://www.ensembl.org/Anopheles_gambiae/contigview?chr=2L&vc_start=40198251&vc_end=40198881) | Ag.2L.2154.0_CDS_at, Ag.2L.1012.0_CDS_at |
| [AGAP006947](http://www.ensembl.org/Anopheles_gambiae/geneview?gene=AGAP006947) |  | [40199453](http://www.ensembl.org/Anopheles_gambiae/contigview?chr=2L&vc_start=40199453&vc_end=40201464) | [40201464](http://www.ensembl.org/Anopheles_gambiae/contigview?chr=2L&vc_start=40199453&vc_end=40201464) | Ag.2L.1462.1_a_at, Ag.2L.2695.0_CDS_a_at, Ag.2L.1462.0_CDS_at |
| [AGAP006948](http://www.ensembl.org/Anopheles_gambiae/geneview?gene=AGAP006948) |  | [40201873](http://www.ensembl.org/Anopheles_gambiae/contigview?chr=2L&vc_start=40201873&vc_end=40207556) | [40207556](http://www.ensembl.org/Anopheles_gambiae/contigview?chr=2L&vc_start=40201873&vc_end=40207556) | Ag.2L.2695.0_CDS_a_at, Ag.2L.1462.0_CDS_at |
| [AGAP006949](http://www.ensembl.org/Anopheles_gambiae/geneview?gene=AGAP006949) | netalloendopeptidase | [40208758](http://www.ensembl.org/Anopheles_gambiae/contigview?chr=2L&vc_start=40208758&vc_end=40211975) | [40211975](http://www.ensembl.org/Anopheles_gambiae/contigview?chr=2L&vc_start=40208758&vc_end=40211975) | Ag.2L.832.0_CDS_at |
| [AGAP006950](http://www.ensembl.org/Anopheles_gambiae/geneview?gene=AGAP006950) |  | [40212417](http://www.ensembl.org/Anopheles_gambiae/contigview?chr=2L&vc_start=40212417&vc_end=40214323) | [40214323](http://www.ensembl.org/Anopheles_gambiae/contigview?chr=2L&vc_start=40212417&vc_end=40214323) | Ag.2L.2155.1_a_at, Ag.2L.2155.2_a_at, Ag.2L.924.0_CDS_a_at |
| [AGAP006951](http://www.ensembl.org/Anopheles_gambiae/geneview?gene=AGAP006951) |  | [40214550](http://www.ensembl.org/Anopheles_gambiae/contigview?chr=2L&vc_start=40214550&vc_end=40215867) | [40215867](http://www.ensembl.org/Anopheles_gambiae/contigview?chr=2L&vc_start=40214550&vc_end=40215867) | Ag.2L.667.0_CDS_at, Ag.2L.924.0_CDS_a_at |
| [AGAP006952](http://www.ensembl.org/Anopheles_gambiae/geneview?gene=AGAP006952) | O-sialoglycoprotein endopeptidase | [40216106](http://www.ensembl.org/Anopheles_gambiae/contigview?chr=2L&vc_start=40216106&vc_end=40217352) | [40217352](http://www.ensembl.org/Anopheles_gambiae/contigview?chr=2L&vc_start=40216106&vc_end=40217352) | Ag.2L.667.0_CDS_at, Ag.2L.1013.0_CDS_at |
| [AGAP006953](http://www.ensembl.org/Anopheles_gambiae/geneview?gene=AGAP006953) |  | [40217380](http://www.ensembl.org/Anopheles_gambiae/contigview?chr=2L&vc_start=40217380&vc_end=40218050) | [40218050](http://www.ensembl.org/Anopheles_gambiae/contigview?chr=2L&vc_start=40217380&vc_end=40218050) | Ag.2L.667.0_CDS_at, Ag.2L.1013.0_CDS_at |
| [AGAP006954](http://www.ensembl.org/Anopheles_gambiae/geneview?gene=AGAP006954) | Clip-Domain Serine Protease CLIPA10 | [40219748](http://www.ensembl.org/Anopheles_gambiae/contigview?chr=2L&vc_start=40219748&vc_end=40224804) | [40224804](http://www.ensembl.org/Anopheles_gambiae/contigview?chr=2L&vc_start=40219748&vc_end=40224804) | Ag.2L.1013.0_CDS_at, Ag.2L.80.0_CDS_at |
| [AGAP006955](http://www.ensembl.org/Anopheles_gambiae/geneview?gene=AGAP006955) |  | [40233778](http://www.ensembl.org/Anopheles_gambiae/contigview?chr=2L&vc_start=40233778&vc_end=40235567) | [40235567](http://www.ensembl.org/Anopheles_gambiae/contigview?chr=2L&vc_start=40233778&vc_end=40235567) | Ag.2L.2696.0_CDS_at |
| [AGAP006956](http://www.ensembl.org/Anopheles_gambiae/geneview?gene=AGAP006956) | carboxylesterase | [40240418](http://www.ensembl.org/Anopheles_gambiae/contigview?chr=2L&vc_start=40240418&vc_end=40252213) | [40252213](http://www.ensembl.org/Anopheles_gambiae/contigview?chr=2L&vc_start=40240418&vc_end=40252213) | Ag.2L.2156.0_CDS_at, Ag.2L.2819.0_at, Ag.2L.626.0_CDS_a_at |
| [AGAP006957](http://www.ensembl.org/Anopheles_gambiae/geneview?gene=AGAP006957) |  | [40253770](http://www.ensembl.org/Anopheles_gambiae/contigview?chr=2L&vc_start=40253770&vc_end=40255412) | [40255412](http://www.ensembl.org/Anopheles_gambiae/contigview?chr=2L&vc_start=40253770&vc_end=40255412) | Ag.2L.2156.0_CDS_at, Ag.2L.626.0_CDS_a_at, Ag.2L.2819.0_at |
| [AGAP006958](http://www.ensembl.org/Anopheles_gambiae/geneview?gene=AGAP006958) | Heat shock protein 83 | [40256208](http://www.ensembl.org/Anopheles_gambiae/contigview?chr=2L&vc_start=40256208&vc_end=40258932) | [40258932](http://www.ensembl.org/Anopheles_gambiae/contigview?chr=2L&vc_start=40256208&vc_end=40258932) | Ag.2L.3183.0_at, Ag.2L.1463.0_CDS_s_at, Ag.2L.283.0_CDS_s_at |
| [AGAP006959](http://www.ensembl.org/Anopheles_gambiae/geneview?gene=AGAP006959) | Heat shock protein 83 | [40260017](http://www.ensembl.org/Anopheles_gambiae/contigview?chr=2L&vc_start=40260017&vc_end=40263217) | [40263217](http://www.ensembl.org/Anopheles_gambiae/contigview?chr=2L&vc_start=40260017&vc_end=40263217) | Ag.2L.1463.0_CDS_s_at, Ag.2L.283.0_CDS_s_at, Ag.2L.2903.0_at, Ag.2L.1464.0_CDS_at |
| [AGAP006960](http://www.ensembl.org/Anopheles_gambiae/geneview?gene=AGAP006960) | tyrosine kinase | [40264493](http://www.ensembl.org/Anopheles_gambiae/contigview?chr=2L&vc_start=40264493&vc_end=40267199) | [40267199](http://www.ensembl.org/Anopheles_gambiae/contigview?chr=2L&vc_start=40264493&vc_end=40267199) | Ag.2L.1464.0_CDS_at, Ag.2L.2157.0_CDS_at |
| [AGAP006961](http://www.ensembl.org/Anopheles_gambiae/geneview?gene=AGAP006961) | Heat shock protein 83 | [40268753](http://www.ensembl.org/Anopheles_gambiae/contigview?chr=2L&vc_start=40268753&vc_end=40271222) | [40271222](http://www.ensembl.org/Anopheles_gambiae/contigview?chr=2L&vc_start=40268753&vc_end=40271222) | Ag.2L.1463.0_CDS_s_at, Ag.2L.283.0_CDS_s_at |
| [AGAP006962](http://www.ensembl.org/Anopheles_gambiae/geneview?gene=AGAP006962) | inositol or phosphoinositol phosphatase | [40275166](http://www.ensembl.org/Anopheles_gambiae/contigview?chr=2L&vc_start=40275166&vc_end=40276976) | [40276976](http://www.ensembl.org/Anopheles_gambiae/contigview?chr=2L&vc_start=40275166&vc_end=40276976) | Ag.2L.2158.1_a_at, Ag.2L.302.0_CDS_at |
| [AGAP006963](http://www.ensembl.org/Anopheles_gambiae/geneview?gene=AGAP006963) | mitochondrial carrier homolog 1 | [40277149](http://www.ensembl.org/Anopheles_gambiae/contigview?chr=2L&vc_start=40277149&vc_end=40278923) | [40278923](http://www.ensembl.org/Anopheles_gambiae/contigview?chr=2L&vc_start=40277149&vc_end=40278923) | Ag.2L.2158.1_a_at, Ag.2L.302.0_CDS_at |
| [AGAP006964](http://www.ensembl.org/Anopheles_gambiae/geneview?gene=AGAP006964) | pyroglutamyol-peptidase 1 | [40279726](http://www.ensembl.org/Anopheles_gambiae/contigview?chr=2L&vc_start=40279726&vc_end=40280593) | [40280593](http://www.ensembl.org/Anopheles_gambiae/contigview?chr=2L&vc_start=40279726&vc_end=40280593) | Ag.2L.1266.0_CDS_at, Ag.2L.302.0_CDS_at |
| [AGAP006965](http://www.ensembl.org/Anopheles_gambiae/geneview?gene=AGAP006965) | TRS85 homolog; ER to Golgi transport | [40281223](http://www.ensembl.org/Anopheles_gambiae/contigview?chr=2L&vc_start=40281223&vc_end=40285900) | [40285900](http://www.ensembl.org/Anopheles_gambiae/contigview?chr=2L&vc_start=40281223&vc_end=40285900) | Ag.2L.1466.0_CDS_at |
| [AGAP006966](http://www.ensembl.org/Anopheles_gambiae/geneview?gene=AGAP006966) | 4-aminobutyrate transaminase | [40286596](http://www.ensembl.org/Anopheles_gambiae/contigview?chr=2L&vc_start=40286596&vc_end=40291145) | [40291145](http://www.ensembl.org/Anopheles_gambiae/contigview?chr=2L&vc_start=40286596&vc_end=40291145) | Ag.2L.344.0_CDS_at |
| [AGAP006967](http://www.ensembl.org/Anopheles_gambiae/geneview?gene=AGAP006967) | inositol or phosphoinositol phosphatase | [40298219](http://www.ensembl.org/Anopheles_gambiae/contigview?chr=2L&vc_start=40298219&vc_end=40368269) | [40368269](http://www.ensembl.org/Anopheles_gambiae/contigview?chr=2L&vc_start=40298219&vc_end=40368269) | Ag.2L.1267.0_CDS_a_at |
| [AGAP006968](http://www.ensembl.org/Anopheles_gambiae/geneview?gene=AGAP006968) | anion exchanger | [40404295](http://www.ensembl.org/Anopheles_gambiae/contigview?chr=2L&vc_start=40404295&vc_end=40407881) | [40407881](http://www.ensembl.org/Anopheles_gambiae/contigview?chr=2L&vc_start=40404295&vc_end=40407881) | Ag.2L.2159.0_CDS_at |
| [AGAP006969](http://www.ensembl.org/Anopheles_gambiae/geneview?gene=AGAP006969) |  | [40410402](http://www.ensembl.org/Anopheles_gambiae/contigview?chr=2L&vc_start=40410402&vc_end=40412690) | [40412690](http://www.ensembl.org/Anopheles_gambiae/contigview?chr=2L&vc_start=40410402&vc_end=40412690) | Ag.2L.1122.0_CDS_at |
| [AGAP006970](http://www.ensembl.org/Anopheles_gambiae/geneview?gene=AGAP006970) | structural constituent of cytoskeleton | [40421708](http://www.ensembl.org/Anopheles_gambiae/contigview?chr=2L&vc_start=40421708&vc_end=40423973) | [40423973](http://www.ensembl.org/Anopheles_gambiae/contigview?chr=2L&vc_start=40421708&vc_end=40423973) | Ag.2L.345.0_CDS_at |
| [AGAP006971](http://www.ensembl.org/Anopheles_gambiae/geneview?gene=AGAP006971) |  | [40426352](http://www.ensembl.org/Anopheles_gambiae/contigview?chr=2L&vc_start=40426352&vc_end=40426837) | [40426837](http://www.ensembl.org/Anopheles_gambiae/contigview?chr=2L&vc_start=40426352&vc_end=40426837) |  |
| [AGAP006972](http://www.ensembl.org/Anopheles_gambiae/geneview?gene=AGAP006972) | structural constituent of pupal cuticle | [40430067](http://www.ensembl.org/Anopheles_gambiae/contigview?chr=2L&vc_start=40430067&vc_end=40430669) | [40430669](http://www.ensembl.org/Anopheles_gambiae/contigview?chr=2L&vc_start=40430067&vc_end=40430669) | Ag.2L.1467.0_CDS_at |
| [AGAP006973](http://www.ensembl.org/Anopheles_gambiae/geneview?gene=AGAP006973) |  | [40432237](http://www.ensembl.org/Anopheles_gambiae/contigview?chr=2L&vc_start=40432237&vc_end=40432754) | [40432754](http://www.ensembl.org/Anopheles_gambiae/contigview?chr=2L&vc_start=40432237&vc_end=40432754) | Ag.2L.1467.0_CDS_at |
| [AGAP006974](http://www.ensembl.org/Anopheles_gambiae/geneview?gene=AGAP006974) | Toll Like Receptor TOLL9 | [40434581](http://www.ensembl.org/Anopheles_gambiae/contigview?chr=2L&vc_start=40434581&vc_end=40440489) | [40440489](http://www.ensembl.org/Anopheles_gambiae/contigview?chr=2L&vc_start=40434581&vc_end=40440489) | Ag.2L.2699.0_CDS_at, Ag.2L.140.0_CDS_at |
| [AGAP006975](http://www.ensembl.org/Anopheles_gambiae/geneview?gene=AGAP006975) |  | [40442431](http://www.ensembl.org/Anopheles_gambiae/contigview?chr=2L&vc_start=40442431&vc_end=40442879) | [40442879](http://www.ensembl.org/Anopheles_gambiae/contigview?chr=2L&vc_start=40442431&vc_end=40442879) |  |
| [AGAP006976](http://www.ensembl.org/Anopheles_gambiae/geneview?gene=AGAP006976) |  | [40447782](http://www.ensembl.org/Anopheles_gambiae/contigview?chr=2L&vc_start=40447782&vc_end=40448369) | [40448369](http://www.ensembl.org/Anopheles_gambiae/contigview?chr=2L&vc_start=40447782&vc_end=40448369) | Ag.2L.1014.0_CDS_at |
| [AGAP006977](http://www.ensembl.org/Anopheles_gambiae/geneview?gene=AGAP006977) |  | [40449192](http://www.ensembl.org/Anopheles_gambiae/contigview?chr=2L&vc_start=40449192&vc_end=40449960) | [40449960](http://www.ensembl.org/Anopheles_gambiae/contigview?chr=2L&vc_start=40449192&vc_end=40449960) | Ag.2L.1014.0_CDS_at |
| [AGAP006978](http://www.ensembl.org/Anopheles_gambiae/geneview?gene=AGAP006978) |  | [40454358](http://www.ensembl.org/Anopheles_gambiae/contigview?chr=2L&vc_start=40454358&vc_end=40454747) | [40454747](http://www.ensembl.org/Anopheles_gambiae/contigview?chr=2L&vc_start=40454358&vc_end=40454747) |  |
| [AGAP006979](http://www.ensembl.org/Anopheles_gambiae/geneview?gene=AGAP006979) |  | [40455588](http://www.ensembl.org/Anopheles_gambiae/contigview?chr=2L&vc_start=40455588&vc_end=40455971) | [40455971](http://www.ensembl.org/Anopheles_gambiae/contigview?chr=2L&vc_start=40455588&vc_end=40455971) |  |
| [AGAP006980](http://www.ensembl.org/Anopheles_gambiae/geneview?gene=AGAP006980) |  | [40457436](http://www.ensembl.org/Anopheles_gambiae/contigview?chr=2L&vc_start=40457436&vc_end=40457812) | [40457812](http://www.ensembl.org/Anopheles_gambiae/contigview?chr=2L&vc_start=40457436&vc_end=40457812) | Ag.2L.2700.0_CDS_at |
| [AGAP006981](http://www.ensembl.org/Anopheles_gambiae/geneview?gene=AGAP006981) |  | [40459508](http://www.ensembl.org/Anopheles_gambiae/contigview?chr=2L&vc_start=40459508&vc_end=40459898) | [40459898](http://www.ensembl.org/Anopheles_gambiae/contigview?chr=2L&vc_start=40459508&vc_end=40459898) | Ag.2L.2700.0_CDS_at |
| [AGAP006982](http://www.ensembl.org/Anopheles_gambiae/geneview?gene=AGAP006982) |  | [40461688](http://www.ensembl.org/Anopheles_gambiae/contigview?chr=2L&vc_start=40461688&vc_end=40462081) | [40462081](http://www.ensembl.org/Anopheles_gambiae/contigview?chr=2L&vc_start=40461688&vc_end=40462081) |  |
| [AGAP006983](http://www.ensembl.org/Anopheles_gambiae/geneview?gene=AGAP006983) |  | [40462641](http://www.ensembl.org/Anopheles_gambiae/contigview?chr=2L&vc_start=40462641&vc_end=40463031) | [40463031](http://www.ensembl.org/Anopheles_gambiae/contigview?chr=2L&vc_start=40462641&vc_end=40463031) |  |
| [AGAP006984](http://www.ensembl.org/Anopheles_gambiae/geneview?gene=AGAP006984) |  | [40464996](http://www.ensembl.org/Anopheles_gambiae/contigview?chr=2L&vc_start=40464996&vc_end=40465372) | [40465372](http://www.ensembl.org/Anopheles_gambiae/contigview?chr=2L&vc_start=40464996&vc_end=40465372) |  |
| [AGAP006985](http://www.ensembl.org/Anopheles_gambiae/geneview?gene=AGAP006985) |  | [40467550](http://www.ensembl.org/Anopheles_gambiae/contigview?chr=2L&vc_start=40467550&vc_end=40467938) | [40467938](http://www.ensembl.org/Anopheles_gambiae/contigview?chr=2L&vc_start=40467550&vc_end=40467938) | Ag.2L.2701.0_CDS_at |
| [AGAP006986](http://www.ensembl.org/Anopheles_gambiae/geneview?gene=AGAP006986) |  | [40470393](http://www.ensembl.org/Anopheles_gambiae/contigview?chr=2L&vc_start=40470393&vc_end=40470890) | [40470890](http://www.ensembl.org/Anopheles_gambiae/contigview?chr=2L&vc_start=40470393&vc_end=40470890) | Ag.2L.2160.0_CDS_at |
| [AGAP006987](http://www.ensembl.org/Anopheles_gambiae/geneview?gene=AGAP006987) |  | [40471592](http://www.ensembl.org/Anopheles_gambiae/contigview?chr=2L&vc_start=40471592&vc_end=40478766) | [40478766](http://www.ensembl.org/Anopheles_gambiae/contigview?chr=2L&vc_start=40471592&vc_end=40478766) |  |
| [AGAP006988](http://www.ensembl.org/Anopheles_gambiae/geneview?gene=AGAP006988) | tyrosine kinase; signal transduction | [40480185](http://www.ensembl.org/Anopheles_gambiae/contigview?chr=2L&vc_start=40480185&vc_end=40482503) | [40482503](http://www.ensembl.org/Anopheles_gambiae/contigview?chr=2L&vc_start=40480185&vc_end=40482503) | Ag.2L.2702.0_CDS_at |
| [AGAP006989](http://www.ensembl.org/Anopheles_gambiae/geneview?gene=AGAP006989) | microtubule motor | [40483191](http://www.ensembl.org/Anopheles_gambiae/contigview?chr=2L&vc_start=40483191&vc_end=40485711) | [40485711](http://www.ensembl.org/Anopheles_gambiae/contigview?chr=2L&vc_start=40483191&vc_end=40485711) | Ag.2L.1724.1_a_at |
| [AGAP006990](http://www.ensembl.org/Anopheles_gambiae/geneview?gene=AGAP006990) | positive regulation of transcription, DNA-dependent; brahma complex | [40485843](http://www.ensembl.org/Anopheles_gambiae/contigview?chr=2L&vc_start=40485843&vc_end=40495867) | [40495867](http://www.ensembl.org/Anopheles_gambiae/contigview?chr=2L&vc_start=40485843&vc_end=40495867) | Ag.2L.1468.0_CDS_a_at, Ag.2L.1725.0_CDS_at |
| [AGAP006991](http://www.ensembl.org/Anopheles_gambiae/geneview?gene=AGAP006991) |  | [40496821](http://www.ensembl.org/Anopheles_gambiae/contigview?chr=2L&vc_start=40496821&vc_end=40497459) | [40497459](http://www.ensembl.org/Anopheles_gambiae/contigview?chr=2L&vc_start=40496821&vc_end=40497459) | Ag.2L.1725.0_CDS_at |

^1^Based on annotation from AgamP3.4 if available, or AnoXcel ver. Feb 2006 (www.vectobase.org).
